# The reduction of DSS-induced colitis severity in mice exposed to cigarette smoke is linked to immune modulation and microbial shifts

**DOI:** 10.1038/s41598-020-60175-3

**Published:** 2020-03-02

**Authors:** Giuseppe Lo Sasso, Blaine W. Phillips, Alain Sewer, James N. D. Battey, Athanasios Kondylis, Marja Talikka, Bjoern Titz, Emmanuel Guedj, Dariusz Peric, David Bornand, Remi Dulize, Celine Merg, Maica Corciulo, Sonia Ouadi, Rendy Yanuar, Ching Keong Tung, Nikolai V. Ivanov, Manuel C. Peitsch, Julia Hoeng

**Affiliations:** 1 PMI R&D, Philip Morris Products S.A., Quai Jeanrenaud 5, CH-2000, Neuchâtel, Switzerland; 2 PMI R&D, Philip Morris International Research Laboratories Pte. Ltd., Science Park II, Singapore

**Keywords:** Molecular biology, Gastrointestinal diseases, Gastrointestinal models

## Abstract

Exposure to cigarette smoke (CS) causes detrimental health effects, increasing the risk of cardiovascular, pulmonary diseases and carcinogenesis in exposed individuals. The impact of CS on Inflammatory Bowel Disease (IBD) has been established by a number of epidemiological and clinical studies. In fact, CS is associated with a higher risk of developing Crohn’s disease (CD) while inversely correlates with the development, disease risks, and relapse rate of ulcerative colitis (UC). To investigate the effect of CS exposure on experimental colitis, we performed a comprehensive and integrated comparative analysis of colon transcriptome and microbiome in mice exposed to dextran sodium sulfate (DSS) and CS. Colon transcriptome analysis revealed that CS downregulated specific pathways in a concentration-dependent manner, affecting both the inflammatory state and composition of the gut microbiome. Metagenomics analysis demonstrated that CS can modulate DSS-induced dysbiosis of specific bacterial genera, contributing to resolve the inflammation or accelerate recovery. The risks of smoking far outweigh any possible benefit, thus smoking cessation must always be encouraged because of its significant health benefits. However, the inverse association between active smoking and the development of UC cannot be ignored and the present study lays the foundation for investigating potential molecular mechanisms responsible for the attenuation of colitis by certain compounds of tobacco when decoupled from combustion.

## Introduction

Inflammatory bowel disease (IBD) is a group of disorders characterized by chronic inflammation of the gastrointestinal tract. Currently, the prevalence of IBD in the Western world is up to 0.5% of the general population, with a steady climb expected over the next decade^1^. Of note, the rising incidence of IBD in newly industrialized countries indicates an emerging epidemic of the disease outside of the Western world^2^. This increasing global burden of IBD is accompanied by a very high economic impact on healthcare systems due to hospitalizations, surgery, ambulatory care, and pharmaceuticals. Furthermore, the indirect costs of IBD, such as loss in work productivity, unemployment, and reduced quality of life, although difficult to estimate, far exceed direct costs, with a heavy impact on society^3^. IBD typically manifests as either ulcerative colitis (UC) or Crohn’s disease (CD); despite some overlap, the two conditions present distinctive clinical and pathological features. No unique cause has been determined for IBD, but its etiopathogenesis is thought to arise from a genetic susceptibility to dysregulated interaction between immune factors and the enteric commensal flora. Environmental triggers, such as drug use, stress, diet, and smoking, influence disease onset and development^4^. The impact of cigarette smoke (CS) on IBD has been established by a large number of epidemiological studies^5^. CS causes detrimental health effects, increasing the risk of cardiovascular and pulmonary diseases and carcinogenesis in exposed individuals^6^. Moreover, in CD patients, smoking is associated with greater disease activity, increased requirement for immunosuppressants, a higher risk of stricturing and fistulizing disease, and early post-operative recurrence^7^. Many of the adverse health effects of smoking are reversible, and important health benefits are associated with smoking cessation^6^ or complementary approaches, as suggested by the tobacco harm reduction initiative^8,9^, to accelerate the decline in smoking prevalence and hence the smoking-related population harm. However, the effects of smoking on UC are unclear. In 1976, Samuelsson first reported a link between smoking and UC in a cohort of UC patients, most of whom were non-smokers^10^. Since then, numerous studies have supported the inverse association between active smoking and the development of UC, including effects on disease risks, progression, relapse rate, and course^11^. While there are multiple epidemiological publications on CS and UC, only a few studies have investigated the mechanistic effect of CS on intestinal inflammation. Several potential mechanisms, including modulation of the mucosal immune response, alterations in intestinal cytokine and eicosanoid levels, and modification of gut permeability, have been proposed. The underlying mechanisms likely involve various smoke constituents, such as nicotine, oxygen free radicals, or carbon monoxide, probably acting on different targets (e.g., mucus layer, gut microbiota, immune cells, gastrointestinal motility, or microvasculature)^12^. Understanding CS effects on intestinal inflammation is challenging; for example, disruption of the gut microbiota equilibrium, termed dysbiosis, is directly linked to dysregulation of the immune response in the gut and is increasingly recognized as a contributing factor in the pathogenesis of IBD^13^. Furthermore, the composition and stability of the mucus layer represents a critical aspect in preventing direct contact between the host and potentially pathogenic bacteria. Of note, each of these components is affected by the inflammatory processes as well as by CS exposure^14^. Both genetic and chemically induced IBD mouse models have been used to elucidate the molecular mechanisms behind CS-IBD interaction. Consistent with findings of epidemiological studies, CS had opposing effects on the development of CD (negatively) and UC (positively) in these models^12,15^. Although CS inhalation studies in mouse models appear to mimic most of the observations in humans, there are differences in the inhalation methodologies, making it difficult to compare the studies^16–19^. Systems-level approaches that leverage omics technologies enable a detailed mechanistic investigation of the disease and treatment effects. For example, we previously used the network perturbation amplitude (NPA) approach with causal network models to stratify the effects of the UC drug infliximab between responders and non-responders from transcriptomic data^20^. Analogously, metagenomics approaches that quantify bacterial populations enable a quantitative characterization of the gut microbiome and alterations in IBD. These approaches will eventually permit a more holistic view of IBD etiology, pathogenesis, and therapy^21^. In the present study, we combined a validated UC mouse model (dextran sulfate sodium, DSS) and a well-controlled inhalation exposure design with disease-specific endpoints and system-wide molecular profiling to investigate the effect of mainstream CS exposure on experimental colitis. While previous publications have assessed the impact of CS on specific cell types, such as immune cells^16,19^, or molecular pathways, such as xenobiotic detoxification enzymes^22^, here we targeted the whole organ using a molecular approach. We leveraged computational analysis of the gut transcriptome and microbiome to identify the molecular mechanisms responsible for the attenuation of mouse colitis by CS at the site of the disease.

Unveiling the molecular mechanisms through which CS exposure alters gene expression and/or intestinal microbiota composition, diversity, and function will advance our understanding of IBD etiology. Data acquired through this study highlight new molecular targets that may represent avenues for preventative strategies and support the potential to develop novel drugs.

## Results

### Test atmosphere generation and aerosol uptake

**Figure 1:**
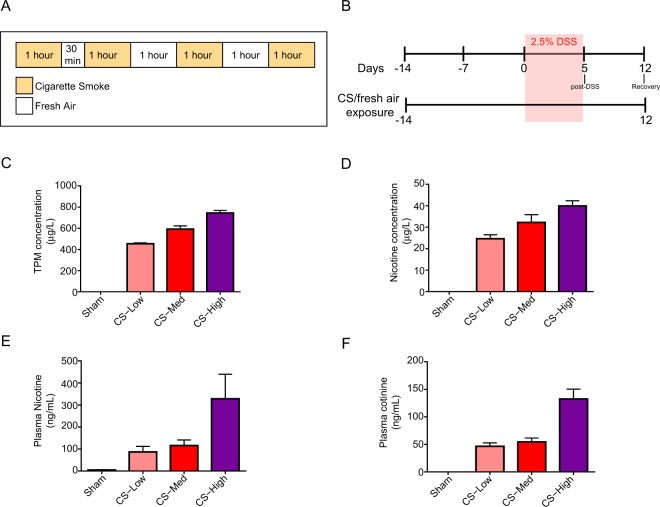
Study design and exposure parameters. (**A**) mice were whole body-exposed to three concentrations (low, medium, and high) of mainstream CS or to fresh air for a total of 27 days for four hours per day interspersed by 30 min or one hour of fresh air. (**B**) DSS treatment in drinking water lasted five days and was followed by seven days of recovery (continued exposure to CS or fresh air). Day 0 represents the starting day for DSS treatment. Post-DSS (day 5) and Recovery (day 12) represent the two time points for sample/data collection. (**C**,**D**) test atmosphere characterization of average TPM (**C**) and nicotine (**D**) levels in the exposure chambers. (**E**,**F**) plasma nicotine (**E**) and cotinine (**F**) concentrations were used as biomarkers of exposure. Data are shown as means ±  SEM. TPM, total particulate matter.

To evaluate the impact of CS on the severity of DSS-induced colitis, mice exposed to CS were treated with 2.5% DSS in drinking water for five days followed by one week of recovery without DSS (Fig. 1A,B).

CS was generated, diluted, and delivered to the exposure chambers in a consistent and reproducible manner. Final average total particulate matter (TPM) values in the CS exposure chambers were 0 (−2.6 µg/L), 454.4 µg/L, 591.8 µg/L, and 744.6 µg/L, all within 5% of the target TPM concentrations (Fig. 1C). These TPM values corresponded to average nicotine concentrations of 0 (below the limit of detection [LOD]), 24.7 µg/L, 32.2 µg/L, and 40.0 µg/L, respectively (Fig. 1D). Carbon monoxide levels in the CS exposure chambers ranged from 513.4 ppm (CS Low) to 802.8 ppm (CS High) and corresponded to the TPM concentrations in each of the chambers (Supplementary Table S1). Levels of the three aldehyde species that are known CS products of combustion were also concentration-dependent in the test aerosols (Supplementary Table S1).

The particle size distribution, measured once per study for each exposure chamber, had a mass median aerodynamic diameter of 0.82–0.85 µm in the CS exposure chambers, with a geometric standard deviation of 1.3–2.5 (Supplementary Table S1). This analysis confirmed the expected particle size distribution to be within a respirable range^23^. Blood nicotine and cotinine levels were concentration-dependent and consistent with levels observed previously in animals exposed to similar concentrations of CS (Fig. 1E,F), confirming aerosol uptake with group uptake levels averaging 86.7 ng/ml, 115.8 ng/ml and 327.7 ng/ml for the low, medium and high exposure groups respectively. While these plasma levels were generally aligned with previously conducted studies in mice using similar exposure nicotine concentrations^24^, the nicotine uptake in the CS High group was nonetheless disproportionately higher than expected (Fig. 1E), which may indicate a change in the respiratory behavior of the animals in this exposure chamber, such as increased minute volume and/or breathing frequency.

### Impact of CS exposure on DSS-induced colitis

**Figure 2:**
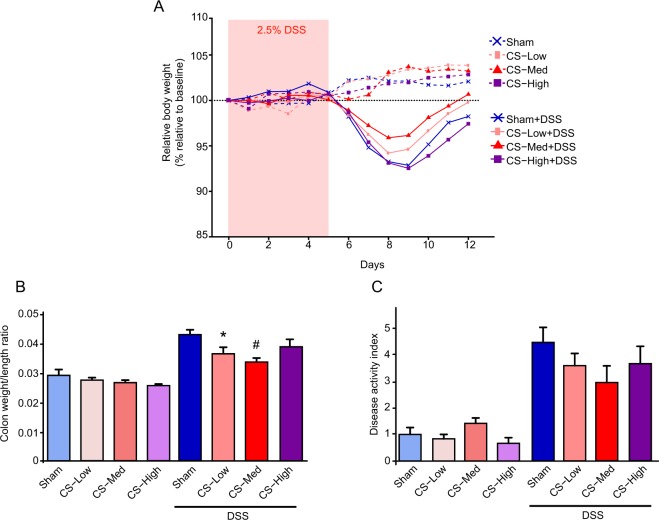
Effect of CS exposure on clinical parameters of DSS-induced colitis. (**A**) changes in mouse body weight during the colitis induction and recovery phases. Weight changes were calculated as a percentage of weight prior to DSS treatment (Day 0). (**B**) the colon weight/length ratio is represented as milligrams per centimeter of colon. (**C**) the DAI was calculated by weight loss, colon weight/length ratio, and stool consistency scoring (Table 2). Data are shown as means  ±  SEM; *p < 0.05, ^#^*p* < 0.01. DAI, Disease Activity Index.

DSS-treated mice developed colitis, as evidenced by weight loss (Fig. 2A), increased colon weight/length ratio (Fig. 2B), and the disease activity index (DAI) (Fig. 2C). CS exposure in mice not subjected to DSS treatment had no significant effects on those parameters. However, animals pre-exposed to CS and concomitantly treated with DSS showed a reduction in colitis severity. In particular, the medium concentration of CS (600 μg/L TPM) improved body weight recovery, prevented colon shortening, and reduced the global DAI (Fig. 2A–C). Histopathological assessment was performed in a separated study focusing only on the CS concentration which was showing higher effectiveness (CS-Medium = 600 μg/L TPM) (Supplementary Fig. S1A–D). The histological scores did not show significant difference between the treatments. These results are in line with previous reports^16,25^, and highlighted the difficulties in capturing histological differences when a recovery period is added; yet other parameters, such as clinical or molecular endpoints can still capture such a differences (see below).

No significant variation in water consumption was registered, indicating that DSS consumption and intake were similar between experimental groups (Supplementary Fig. S2A). Of note, the borderline significance in weight loss and DAI (Fig. 2A,C) became stronger when a single mouse in the CS Medium group was excluded from the statistical analysis (Supplementary Fig. S2B,C). To further characterize the ongoing inflammation, 14 cytokines and chemokines in the distal colon were analyzed (Supplementary Fig. S3 and Table S2). As expected, after one week of recovery without exposure to DSS, levels of multiple cytokines were below the limit of detection (LOD). However, the pro-inflammatory neutrophil chemokine keratinocyte chemoattractant and the cytokines IL-6 and G-CSF showed a trend toward reduction in mice exposed to both CS and DSS but not in mice treated with DSS without CS exposure. The stronger effect was registered in mice exposed to the medium concentration of CS, confirming previous observations. Taken together, these results suggest that CS exposure limited colonic inflammation and/or facilitated recovery from inflammation induced by DSS.

### CS attenuates DSS-induced gene expression changes in the colon

**Figure 3:**
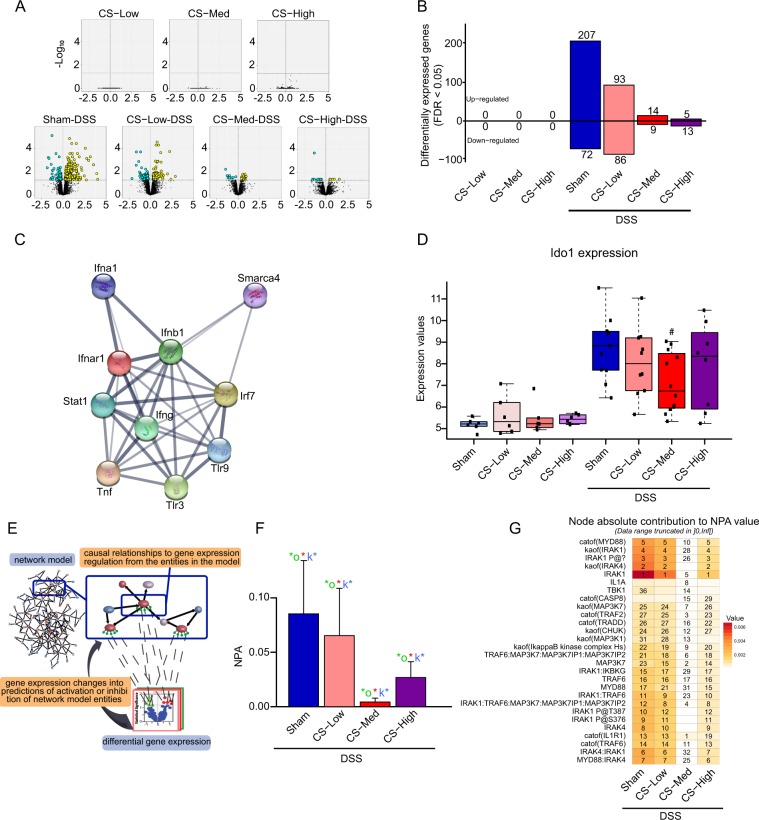
Results of colon transcriptomics analysis. (**A**) volcano plots displaying the amplitude and significance of gene expression changes in the colon for the relevant pairwise comparisons. Gene differential expression (log_2_ fold change) are plotted along the *x*-axis while their statistical significance (log_10_ FDR) is plotted on the *y*-axis. Yellow and blue dots highlight genes that are differentially up- or downregulated, respectively (FDR < 0.05) following exposure to CS (low, medium, or high concentration) with and without DSS treatment. (**B**) bar plot of the number of DEGs (FDR < 0.05). (**C**) IPA UR analysis. The network of the top 10 URs (ranked by z-score) is based on the association confidence provided by StringDB. (**D**) boxplot of the experimental group-based distributions of Ido1 expression in the mouse colon. The distributions are summarized using the standard R convention: thick line = median, box height = interquartile range, whiskers positions = most extreme values or quartiles ± 1.5 × interquartile range. # means raw *p*-value for CS-Med-DSS versus Sham-DSS < 0.05. (**E**–**G**) NPA analysis of DEGs in the colons of DSS- and CS-treated mice. (**E**) network scoring principle for transcriptomic data. (**F**) NPA scores for the TLR-IL1R-TNFR network model are shown with their confidence intervals accounting for experimental variation. Companion statistics derived to inform on the specificity of the NPA score with respect to the biology described in the subnetwork model are shown as *O and K* if their *p*-values are below the significance level. (**G**) an extract of the TLR-IL1R-TNFR pathway showing the most affected entities in the model. The nodes of the network that were the top contributors to the impact were prioritized and interconnected. Each node contains the NPA score for colon samples from mice treated with DSS (1), DSS and 450 µg/L CS (2), DSS and 600 µg/L CS (3), and DSS and 750 µg/L CS (4). Blue bars indicate inferred downregulation, and orange/red bars indicate inferred upregulation. The green asterisk indicates that the node is a leading node in a given contrast. The vocabulary for the Biological Expression Language is provided in http://www.openbel.org/. NPA, Network Perturbation Amplitude.

We performed a global transcriptome analysis of the distal colon to identify biologically relevant effects of CS exposure. First, we compared the amplitudes and significance of gene expression changes in the distal colon of CS-exposed mice with those of the Sham group for each CS concentration (Fig. 3A), in the absence (upper panels) or presence (bottom panels) of DSS. CS exposure resulted in a decrease in the number of differentially expressed genes (DEGs) in the colon of DSS-treated mice (Fig. 3B). No DEGs were identified in colon of DSS-untreated mice exposed to any of the three CS concentrations. Next, to obtain insights into the molecular mechanisms, we conducted an Upstream Regulator (UR) analysis using Ingenuity Pathways Analysis (IPA) tool to identify candidate genes that may drive the transcriptional changes observed^26^. In the Sham-DSS versus Sham comparison, the top 10 predicted upregulated URs ranked by z-score were IFNγ, IRF7, IFNα, Stat1, TNF, INFB1, TLR9, TLR3, IFNAR1, and SMARCA4 (Fig. 3C and Table 1), all known contributors to inflammatory responses. When mice exposed to CS and treated with DSS were compared with Sham-exposed mice, these URs were less or not differentially expressed (Table 1). Of note, the top upregulated gene driving the activation of all of these URs in the Sham-DSS group was indoleamine 2,3-dioxygenase 1 (Ido1). In the DSS-treated group, CS exposure reduced Ido1 upregulation in a concentration-dependent manner (Fig. 3D). The validity of this observation was confirmed by RT-qPCR quantification for a subset of samples (Supplementary Fig. S4A). In DSS-treated mice Ido1 gene levels were increased, however the co-exposure of mice to CS at medium concentration significantly reduce this effect. Further Canonical Pathways analysis via IPA established “Activation of IRF by Cytosolic PRRs” and “Interferon Signaling” as the top two canonical pathways (ranked by *p*-value) activated by DSS specifically in Sham + DSS mice when compared with the Sham group. These two pathways were tightly connected to the TLR signaling pathway via sharing specific target gene, such as STAT2, IRF1, IRF7, IRF9, TAP1, IFI35, OAS2, and PSMB8 (Supplementary Fig. S4B,C). Of note, upon CS exposure these pathways are all switched off, which makes these findings consistent with the observed reduced colonic inflammation.

**Table 1:** Ten most upregulated URs ranked by z-score. *p*-value as well as the number of DEGs contributing to the URs are displayed. DSS, dextran sulfate sodium; CS Low, 450 μg/L TPM from CS; CS Med, 600 μg/L TPM from CS; CS High, 750 μg/L TPM from CS.

Top 10 UR	*p*-value	Sham + DSS	CS Low + DSS	CS Med + DSS	CS High + DSS
		Number of differentially expressed genes			
INFγ	2.03E-25	69	18	3	0
IRF7	2.12E-24	36	7	0	0
INFα	1.35E-21	38	6	0	0
Stat1	4.23E-34	46	8	1	0
TNF	2.62E-07	47	13	1	0
IFNB1	1.70E-19	30	5	0	0
TLR9	1.14E-12	21	7	0	0
TLR3	1.15E-13	23	5	0	0
IFNAR1	1.19E-21	25	6	0	0
SMARCA4	5.19E-04	18	6	0	0

To obtain more mechanistic detail into how the TLR signaling was affected by CS in the DSS-treated mice, we used a causal biological network model that was constructed previously to assess the response of UC subjects to anti-TNF therapy^20^. This network model contains the TLR receptor, IL-1 receptor 1 (Il1r1), and TNFα receptor signaling branches that lead to NF-κB activation. Figure 3E–G show the scoring of the TLR-IL1R1-TNFR network model with transcriptomic data from colons of mice treated with DSS with and without CS exposure. Exposure to all CS concentrations attenuated the colonic effects of DSS in the context of the network model (Fig. 3F). Evaluation of the most affected entities in the network model revealed that DSS treatment mostly upregulated the IRAK/MYD88 pathway and that this inferred activation was attenuated in response to CS, with the strongest effect observed in mice exposed to the medium CS concentration (Fig. 3G).

### GSA supports the attenuating effect of CS on DSS-induced inflammation

**Figure 4:**
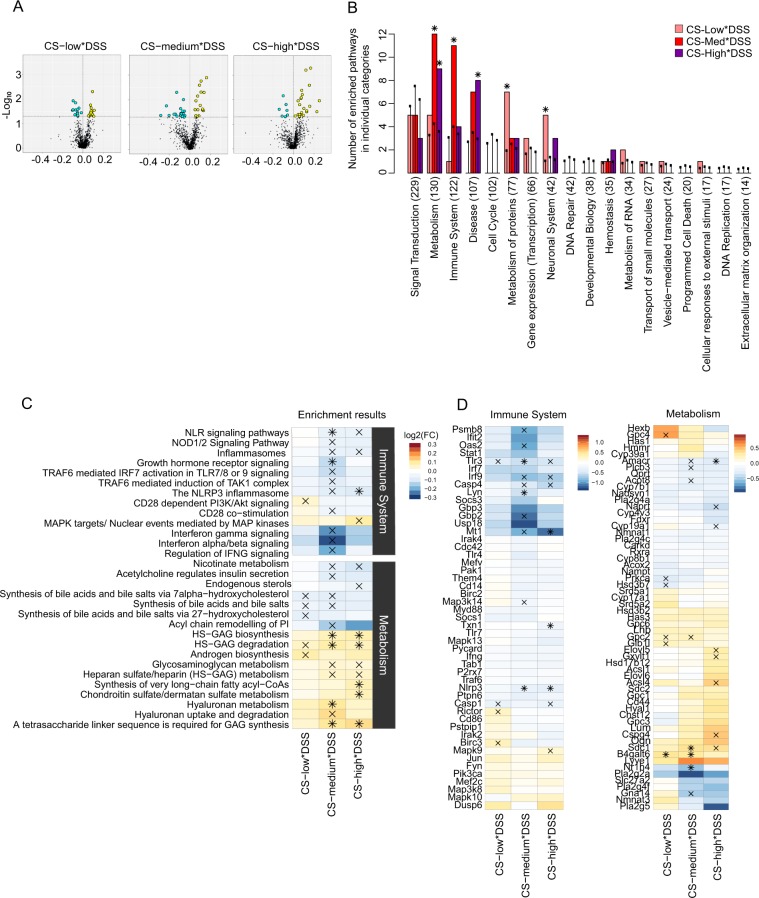
Results of GSA for two-factor statistical models. (**A**) volcano plots displaying the amplitude and significance of the of colon gene set-level response for the interactions (or difference of differences) between DSS treatment and CS exposure (three concentrations). GSA scores are plotted along the *x*-axis while their statistical significance (GSA Q1 *p*-values) is given along the *y*-axis. Yellow and blue dots highlight gene sets that are significantly enriched by up- or downregulated expression, respectively (Q1 *p*-value <0.05). (**B**) over-representation analysis of the significantly enriched gene sets using the Reactome top pathway categories. The colored bars represent the number of enriched gene sets in each pathway category, while the black points indicate the corresponding expected values, based on a random distribution of the enriched gene sets into the pathway top categories. Black plus marks (+) indicate statistically significant differences between the observed and expected numbers of enriched gene sets (over-representation analysis *p*-value <0.05) and show that the “Immune System” and “Metabolism” categories were significantly enriched for mice exposed to the medium concentration of CS. (**C**) heatmap of individual GSA score (DSS treatment and CS exposure interaction) for the pathways in the over-represented Reactome pathway top categories “Immune System” and “Metabolism”. The annotations (+ and *) indicate statistical significance according to GSA Q1 *p*-values (only pathways which display statistical significance at least one of the columns have been included). (**D**) heatmaps of gene differential expression values for the DSS treatment and CS exposure interactions. The genes were obtained from the union of all the leading-edge genes belonging to the statistically significant pathways (according to GSA Q1 *p*-values) contained in the over-represented Reactome top pathway categories “Immune System” and “Metabolism”, respectively. The annotations (+and *) indicate statistically significant gene differential expression according to raw *p*-values.

To confirm the role of CS in the modulation of the identified URs and their related pathways in DSS-induced inflammation, we performed GSA using a statistical model focused directly on the modulating effects of CS exposure on DSS-induced IBD (Supplementary Fig. S5A). CS exposure significantly modulated approximately 40 gene sets (raw *p*-value <0.05) (Fig. 4A). To determine whether these gene sets represented specific biological categories, we performed an over-representation analysis at the gene set level. Among the five significantly enriched categories, “Immune System” and “Metabolism” displayed the strongest impact for the medium concentration of CS (Fig. 4B,C and Supplementary Fig. S5B). The gene sets in the immune system category were mainly linked to pattern recognition receptors, such as TLRs and nucleotide-binding oligomerization domain-like receptors, and IFN α/β/γ signaling (Fig. 4C, upper panel), with several genes contributing to these differences (Fig. 4D, left panel). These observations further support our conclusion that the medium concentration of CS attenuated DSS-dependent inflammatory effects, mainly acting via pattern recognition receptors and IFN signaling. CS exposure also modulated metabolism gene sets, represented by two main groups regulated in opposite directions (Fig. 4C, lower panel), with several “leading-edge” genes inducing these differences (Fig. 4D, right panel). Synthesis of bile acids was downregulated, while glycosaminoglycan and hyaluronan uptake, metabolism, and degradation were upregulated (Fig. 4C,D). To validate the observations obtained by microarray transcriptomics, we used RT-qPCR to quantify the expression levels of a subset of the “leading-edge” genes extracted from both “Immune System” and “Metabolism” categories (Supplementary Fig. S6A,B). The genes were selected among the ones affected by the medium concentration of CS in the presence of DSS (Fig. 4D). RT-qPCR results confirmed the microarray values, with several genes, such as Interferon regulatory factor 9 (Irf9), Guanylate-binding protein 2 (Gbp2), Proteasome subunit beta type-8 (Psmb8), and Beta-1,4-galactosyltransferase 6 (B4galt6) showing statistically significant effects. Globally, the validity of the microarray data was confirmed by the strong correlation values (Pearson coefficient>0.85) observed between the differential gene expressions captured by RT-qPCR and microarray in the different exposure settings (Supplementary Fig. S6C).

Overall, GSA and RT-qPCR results confirmed the quantitative and mechanistic findings from UR analysis and network enrichment, supporting the conclusion that the medium concentration of CS ameliorates DSS-induced inflammatory changes in the murine colon. The modulation of metabolic pathways, such as bile acid synthesis and glycan metabolism, may indicate an interaction with the gut microbiota.

### CS affects gut microbiota composition

Shotgun sequencing of the metagenome was used to investigate CS-related alterations in microbial community composition in a longitudinal manner. Using our combination of DNA isolation, sequencing, and sequencing read taxonomic assignment, the most abundant taxa identified were all bacterial, and thus in the following, only the bacterial abundance shifts will be discussed.

**Figure 5:**
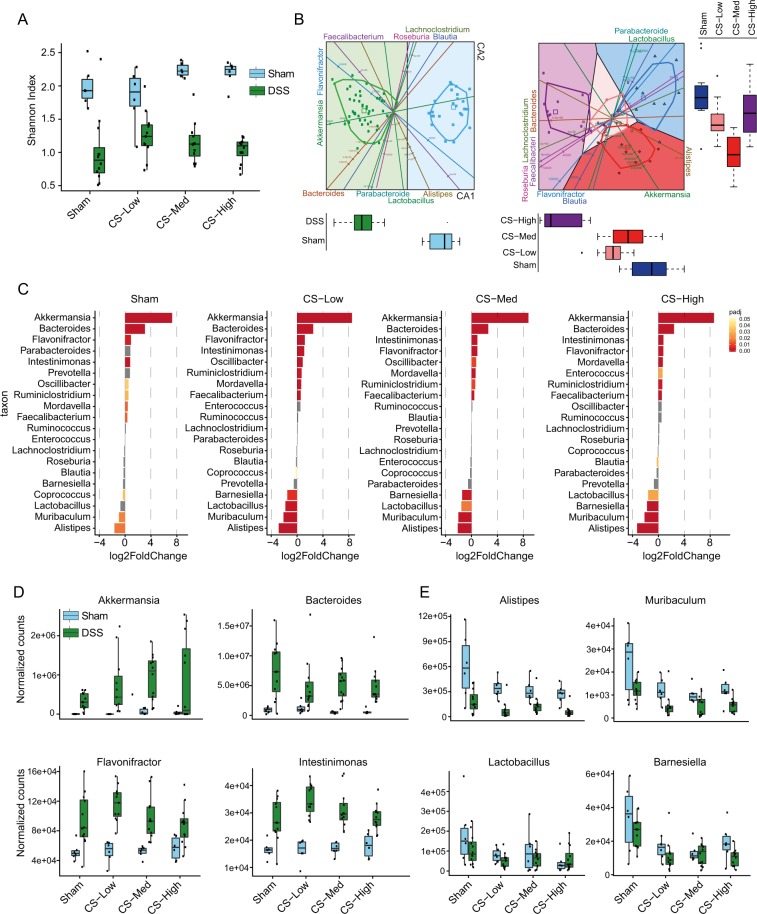
Results of metagenomics analysis post-treatment with DSS. (**A**) alpha diversity (Shannon index) was calculated at the genus level, and is displayed as a bar-and-whiskers plot for each individual combination of DSS treatment status and CS exposure. The center line represents the median and the box encloses the 1st and 3rd quartiles (“hinges”). The upper and lower whiskers represent the furthermost points from the respective hinges, which are no more than 1.5 interquartile ranges from the hinge. The individual points are overlaid. (**B**) canonical variate analysis biplots. Samples (points) and bacterial taxa (axes) are simultaneously projected on the two-dimensional canonical axes (CA1 and CA2). Left panel: plot of the first two canonical variates related to the treatment group; water (no DSS) is depicted in light blue, while DSS treatment is given in green. Right panel: plot of the first two canonical variates related to smoking group in DSS-treated mice. Both panels: thick unfilled squares are used to illustrate the group centroids (canonical means) by the colors codes described above. Thick solid lines colored by group depict the 0.95-bags for each group, reflecting the deepest 95% core of the data for a given group. The original variable (taxon) axes are illustrated simultaneously with the samples to emphasize the correlation between samples and variables (taxa). Axis tick marks and printed tick mark values depict the levels of the original variables (read counts per taxon). Colored regions depict the classification regions by study group. Boxplots are used in the extreme margins to summarize group differences for each canonical variate dimension (horizontal for the first and vertical for the second) and colored according to study group. Color codes and symbols: light blue triangle, Sham; light red squares, low CS; red diamonds, medium CD; violet bullets, high CS. (**C**) differential abundance of bacterial genera for each combination of DSS treatment and CS exposure relative to the untreated and unexposed reference group (Sham). The top 20 most abundant genera determined by the Deseq. 2 BaseMean metric are reported for each comparison. Red stars indicates the top 10 most abundant genera. Bars are colored by differential abundance (adjusted *p*-values [padj] <0.05); grey bars: padj> 0.05. (**D**) normalized read count for the four bacterial genera with greatest increases in abundance. (**E**) normalized read count for the four bacterial genera with greatest decreases in abundance. The bar-and-whisker plots in (**D**,**E**) were constructed as described for A.

Analysis of the Shannon index at the genus level showed the expected reduction in bacterial species richness in the feces of DSS-treated mice on Day 5 post-DSS treatment (Fig. 5A). CS exposure in DSS-treated mice slightly increased species diversity. At the recovery time point (Day 12 in Fig. 1B), the original diversity was partially restored (Supplementary Fig. S7A). For the group exposed to the medium concentration of CS, the interquartile range of the Shannon index distribution overlapped between DSS-treated and untreated animals, suggesting a faster recovery for this exposure group. Using canonical variate analysis, we extracted bacterial genera profiles (reflected by the canonical variate components) that best separated the study groups (DSS and CS exposure; Fig. 5B). Confirming previous reports^27,28^, increases in *Akkermansia*, *Bacteroides*, and *Flavonifractor* permitted a clear separation between DSS-treated and untreated mice on the first canonical axis (Fig. 5B, left panel). Interestingly, CS exposure in DSS-treated mice also confirmed differential bacterial communities among the smoking groups, enabling further discrimination between the CS-exposed mice and the Sham group (Fig. 5B, right panel). The genera *Bacteroides* and *Flavonifractor* best separated the CS-High group from the Sham group on the first canonical axis, while the increased *Akkermansia* observed abundance following CS exposure appeared to drive the separation of the CS-Low and especially CS-Medium groups from the Sham group on the second canonical axis. To quantify the CS-related changes in the microbiota composition of DSS-treated mice, the differential abundance of bacterial genera was calculated. The 20 most abundant genera and their differential abundance with respect to the Sham group (no DSS and no CS) are listed for both post-DSS and recovery time points in Fig. 5C and Supplementary Fig. S7B, respectively. *Akkermansia* (Verrucomicrobia), *Bacteroides* (Bacteroidetes), *Flavonifractor* (Firmicutes), and *Intestinimonas* (Firmicutes) abundance increased significantly in relative abundance following DSS treatment (Fig. 5C, left panel). CS exposure in DSS-treated mice was associated with a further increase in *Akkermansia*, a genus of mucin-degrading bacteria that have been shown previously to be inversely correlated with the onset of inflammation^29^ (Fig. 5D, upper left panel). A similar CS-dependent trend, although less pronounced, was observed for *Flavonifractor* and *Intestinimonas* (Fig. 5D, bottom panels). Finally, among the genera whose abundance increased following DSS treatment, *Bacteroides* exhibited a lower relative abundance in mice exposed to CS (Fig. 5D, upper right panel). In contrast, *Alistipes* (Bacteroidetes), *Muribaculum* (Bacteroidetes), *Lactobacillus* (Firmicutes), and *Barnesiella* (Bacteroidetes) abundance was reduced by DSS treatment (Fig. 5C, left panel). CS exposure slightly reduced their relative abundance in both DSS-treated and untreated animals (Fig. 5E). After seven days of recovery, the relative abundance of the majority of the genera did not differ significantly from that of the Sham group (no DSS and no CS), confirming that the recovery phase paralleled the reduction in the degree of dysbiosis (Supplementary Fig. S7B). *Bacteroides* remained heavily over-abundant, even following recovery, and were not affected by CS exposure (Supplementary Fig. S7B,C, upper left panel). CS exposure did not appear to affect the differential abundance of the majority of the bacterial genera at this later stage (Supplementary Fig. S7C,D). However, it is worth noting the opposing trends displayed by *Akkermansia* and *Lactobacillus* following CS exposure (Supplementary Fig. S7C). The formerly pronounced, statistically significant difference in *Akkermansia* abundance between CS-treated and Sham-DSS mice (Fig. 5C) became less pronounced and was only significant in the CS-Low group (Supplementary Fig. S7B). However, *Akkermansia* abundance at the recovery stage remained higher in the DSS-treated group (Supplementary Fig. S7C, upper right panel). *Lactobacillus* abundance peaked during the DSS recovery period and was reduced by CS in a concentration-dependent manner (Supplementary Fig. S7B, and C bottom left panel). This finding may be attributable to the direct interference of CS with *Lactobacillus* growth. To further confirm the validity of our observations, the differential abundance of specific taxa was measured by qPCR in selected samples from post-DSS time point (Supplementary Fig. S8A,B). The measured relative abundance of each taxon confirmed the previously observed variation upon DSS treatment and exposure to CS at medium concentration, especially for *Akkermansia* and *Barnesiella* (Supplementary Fig. S8A). Of note, correlation of the abundances derived from qPCR and from DNA-Seq data for all samples in this analysis confirmed the robustness of our analysis, particularly for *Akkermansia*, *Lactobacillus, Barnesiella* and *Bacteroides* (Supplementary Fig. S8B). In the case of *Intestinimonas*, the correlation was low; the primer used in this case was designed for a species of *Intestinimonas*, rather than the whole genus, which may explain the mismatch.

Overall, these data show that CS exposure may influence the severe dysbiosis induced by DSS acting on specific bacterial genera, contributing to resolve the inflammation or accelerate the healing process.

### Mucin expression pattern is altered following DSS treatment and CS exposure

**Figure 6:**
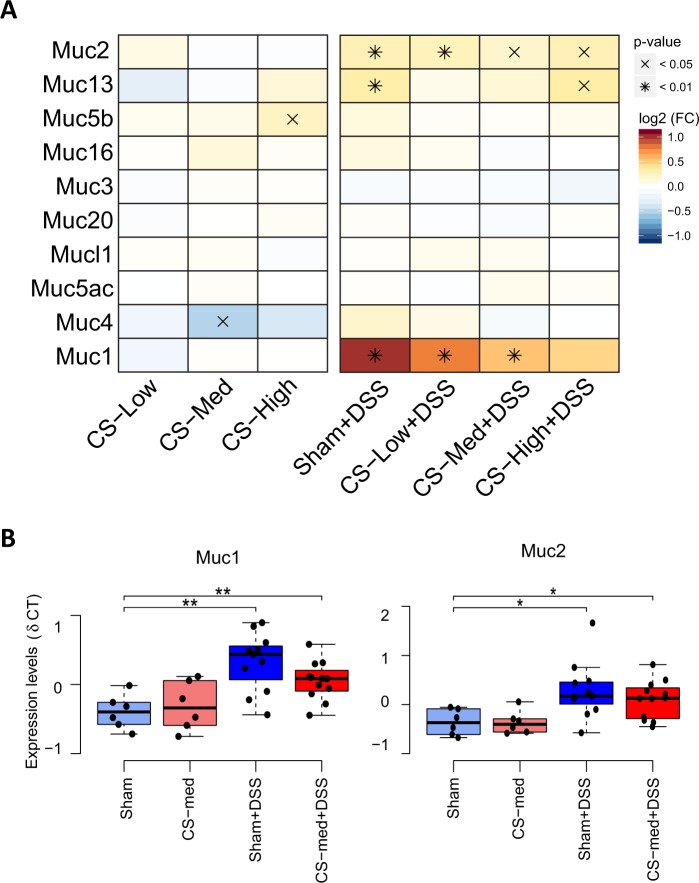
Expression levels of the mucin transcripts that were differentially expressed in the murine distal colon following recovery from DSS treatment and exposure to low, medium, or high concentrations of CS. (**A**) heat map of the microarray-based differential expression values using an intensity-dependent color map and complemented by their statistical significance (raw *p*-value, moderated *t* statistics). The pairwise comparisons are identical to the ones shown in Fig. 3B–D and explained in Supplementary Fig. S5A. FC, fold change. (**B**) box plot of the experimental group-based distributions of Muc1 and Muc2 RT-qPCR-based expression levels in the mouse colon, as quantified by RT-qPCR. The same conventions were used as in Fig. 3D and the horizontal brackets indicate the statistical significance of the corresponding comparisons (* and ** mean *p*-value smaller than 0.05 and 0.01, respectively).

The detected bacterial changes and the CS effect on one of the most abundant mucolytic genera, *Akkermansia*, prompted us to investigate whether mucin gene expression was altered following DSS treatment and/or CS exposure. Figure 6A displays a heatmap of the mucins expressed in the colon at the end-point (Recovery). CS exposure affected *Muc5b* and *Muc4*, which were upregulated and downregulated, respectively (Fig. 6A, left panel). DSS treatment induced a general upregulation in mucin gene expression, as observed in the Sham-DSS group (Fig. 6A, right panel). CS exposure in the DSS group decreased the expression of few mucin genes (*Muc13*, *Muc16*, *Muc4*, *Muc2* and *Muc1*) compared with the Sham-DSS group. Muc1 and Muc2 gene expression was also quantified by RT-qPCR in a subset of samples, confirming the trends induced by DSS and CS (Fig. 6B).

Altogether, we found a few CS-specific effects on mucin gene expression, but at this stage, we cannot determine the real cause-effect link between mucin, the gut microbiota, and inflammation.

## Discussion

The basic molecular etiology of the two IBD manifestations, CD and UC, is still unclear, and the complexity of the diseases increases considerably with contributions of environmental factors. Cigarette smoke (CS) is a well-known and preventable risk factor of multiple diseases, such as cancer and cardiovascular and pulmonary diseases^6^. Many of the adverse health effects of smoking are reversible, and smoking cessation must always be encouraged because of its significant health benefits^30^. CS is considered a relevant environmental modulator of IBD and, surprisingly, affects the two IBD conditions diametrically. CS has been shown to accelerate the clinical progression of CD and increase the risk of disease onset^7^, while reducing the occurrence, progression, and severity of UC^31,32^. Despite the epidemiological, clinical, and pre-clinical attempts to elucidate the molecular mechanisms underlying the inverse correlation between CS and UC, there are still substantial gaps in our knowledge.

To address the correlation between smoking, intestinal inflammation, and gut microbiota composition, we combined a robust mouse UC model^33^ with a well-controlled state-of-the-art inhalation exposure design and technology. Disease-specific endpoints were investigated using a diverse and integrated comparative analysis of the transcriptome and microbiome at the site of the disease. We found that exposure to targeted concentrations of CS reduced the severity of colitis in DSS-treated mice by acting on two separate, but probably interconnected, targets: inflammation and gut microbiota composition.

Only a few studies have used CS exposure in a similar mouse model to investigate this topic^16,19,34,35^, reporting conflicting results and high methodological heterogeneity. This is particularly true for CS exposure schedules and durations as well as for documentation of meaningful concentration measurements in the exposure atmospheres or in animal biofluids^15^. To overcome these limits, we first adapted our study design and inhalation exposure methodology to conform to Organisation for Economic Co-operation and Development (OECD) guidelines for inhalation toxicity studies^23,36^, with detailed characterization of the test atmosphere and biomarkers of exposure.

We used three exposure concentrations, corresponding to human equivalent doses of 40.6 mg, 51.1 mg, and 64.4 mg of nicotine delivery, which correspond respectively to 29, 37, and 46 cigarette sticks (assuming 1.39 mg nicotine per stick)^37^ (see Materials and Methods). The exposure parameters confirmed the tolerability of the CS concentrations we used and applicability to human exposure, providing a clear methodological template for future IBD inhalation studies.

To better recapitulate a human IBD-like condition, we slightly modified the classical 7day-DSS model. Indeed, both CD and UC are relapsing and remitting inflammatory disorders. To model this condition in mice, a 7-day recovery period was included following the 5 day DSS treatment period, thus, by taking the mice off of DSS and administering water during the recovery, we could model the injury and recovery periods seen in human disease. As shown by others^38^, this modified protocol permits the analysis of the immune response shifts from the pro-inflammatory milieu during the acute phase to the anti-inflammatory and pro-wound healing milieu during the recovery phase^39^. Moreover, recent publications have highlighted that, in the context of microbiota analysis, acute DSS colitis is different from IBD while some similarities are often associated to the recovery phase^28^.

Analysis of disease-specific endpoints revealed that exposure to the medium concentration (600 μg/L TPM, corresponding to 32 μg/L nicotine) resulted in a clear reduction in colitis severity. Of note, while the low concentration of CS exerted similar although weaker effects, the higher concentration did not affect disease-specific endpoints. Interestingly, in the absence of inflammation (no-DSS groups), no CS-dependent changes have been observed, which indicate that, in our experimental condition, CS exposure does not affect the epithelium in a pre-inflammatory state.

These observations were confirmed by a first-tier gene expression profiling analysis. The transcriptional network of inflammatory responses in the colon was downregulated in mice exposed to the medium concentration of CS. We identified the predicted top 10 molecules responsible for the activation of inflammatory pathways in the DSS-treated mice that were not exposed to CS. The top upregulated gene driving these effects was Ido1. Exposure to the medium concentration of CS attenuated the upregulation of Ido1 following DSS treatment. A previous study reported that inhibition of Ido1 and consequent downregulation of TLR and NF-κB signaling pathways attenuates the severity of DSS-induced colitis and modulates the inflammatory response^40^. However, in our study, CS modulated Ido1 expression only during the inflammatory period, suggesting an indirect effect due to the lower inflammatory state induced by CS rather than a direct CS-mediated effect on Ido1 expression.

The attenuation of inflammation by CS exposure in DSS-treated mice was further validated by causal biological network modeling^20^ and GSA, supporting a primary role for the TLR and IFN pathways in DSS-induced inflammation^41,42^ and highlighting the CS-dependent attenuation of those pathways. Of note, the upregulation of STAT2, IRF1, TAP1, IRF9, IFI35, and PSMB8 (Supplementary Fig. S4) triggered both the IFN and TLR pathways only in mice treated with DSS but not CS, which in turn prevents the activation of the same target genes. The role of TLR signaling in IBD has been well described. In UC subjects the expression patters of individual TLRs vary depending on the receptor subtype and disease state (quiescent vs active^43^), and TLR gene mutations and functional dysregulation are thought to be the predominant contributors to IBD susceptibility. Moreover, TLRs play an important role in the gut by modulating the mucosal immune responses^44^.

To determine which molecular pathway is directly targeted by CS in the context of DSS-induced inflammation results quite difficult because of the complex mixture of components that constitute CS. This limitation was common to most of the previously published manuscript focusing on the role of CS in the context of DSS-induced colitis. Indeed, two of the most recent publications, Montbarbon *et al*.^16^ and Daniluk *et al*.^19^ have nicely shown the CS-dependent modulation of different immune cell components, e.g. iNKT lymphocyte, CD4^+^ T cell and B cell subpopulation, linking this changes to the different inflammatory states. However, the drivers for these changes are unknown today. Of note, the only pure mechanistic insights in this context have been obtained by treating either mice or cell lines with component of CS, such as nicotine, cotinine, or carbon monoxide. Indeed, a co-culture of Caco2 and enteric glial cells (EGC) barrier breakdown via Cytomix^45^, showed nicotine-dependent improvements by preventing the activation of the nuclear factor kappa-B (NF-κB)-dependent inflammatory pathway. Similarly, Aldhous *et al*.^46^ reported that CS extract treatment of intestinal cell lines delayed TNF-α-induced NOD2 mRNA expression and reduced chemokine production by inhibiting upstream NF-κB translocation. Moreover, the “nicotinic anti-inflammatory pathway”, has been often suggested as a possible mechanism. Studies have demonstrated that in macrophages, involvement of Jak2 kinase activating STAT3 by phosphorylation and pSTAT3, can induce expression of anti-inflammatory proteins such as SOCS3 and prevent NF-κB activation, enhancing the anti-inflammatory effect^47^. In a mouse model of colitis, it was shown that pretreatment with nicotine can reverse the inflammatory effects through the activation of α7nAChR, which was identified as the main receptor involved in the nicotinic anti-inflammatory pathways^48^. Finally, a very recent study by Maruta *et al*.^49^ supported the anti-inflammatory role of nicotine, hypothesizing a nicotine-dependent suppression of MAdCAM-1, a cell adhesion molecule, in the gut micro-vessels. Other CS component have shown anti-inflammatory capabilities, such as carbon monoxide^50^ or other alkaloids like anatabine^51^. Taken together, all these studies highlight how treatment with single constituents of CS, in particular nicotine, facilitates the dissection of more specific molecular mechanism.

Interestingly, a second-tier gene expression profiling via GSA, revealed a CS-dependent regulation of gene sets involved in bile acid synthesis and glycosaminoglycan metabolism. These two metabolic processes share important commonalities and are interconnected in the context of intestinal inflammation, being involved in IBD development and etiology^52,53^, affected by CS^54–56^, and regulated or modulated by the bacterial community dynamics^53,57^. Murine and human studies have suggested that smoking affects the composition of the intestinal microbiome, with subsequent alterations in the pathophysiology of several diseases, such as IBD^58^. However, differences in the methods and animal models used, confounding factors, and no identification of the mechanisms underlying smoking-induced microbial alterations have generated contradictory results. Here, we used shotgun sequencing of the fecal metagenome to investigate possible connections between CS exposure, the gut microbiome, and the reduced severity in colitis. In line with previous observations, DSS-induced inflammation affected the gut microbiota substantially, reducing overall diversity as modulating the abundance of specific genera. *Akkermansia*, *Bacteroides*, and *Flavonifractor* were the most abundant genera, and their abundance increased further with DSS treatment^27,59,60^. In contrast, *Alistipes*, *Muribaculum*, and *Lactobacillus* abundance decreased following DSS treatment^27,61,62^. Analysis of the CS-dependent shifts in gut microbiome composition in DSS-treated mice yielded interesting results. Previous observations showed that Clostridia were differentially affected by CS exposure^34,63^. In agreement with these reports, we observed a CS-dependent effect on genera belonging to the Clostridia class, such as *Flavonifractor* and *Intestinimonas*, the abundance of which increased at the post-DSS time point. *Intestinimonas*, a newly described bacterial genus present in the intestinal tract of humans and other animals^64^, has been shown to be involved in butyrate production and to be affected by bile acid alterations in an obese rat model^64,65^. Seven days after DSS treatment, the relative abundance of other genera of the Clostridia class, such as *Lachnoclostridium*, *Blautia*, *Roseburia*, and *Coprococcus*, decreased in the DSS-treated group and was not significantly affected by CS. We also found that genera belonging to the Bacteroidia class were affected by CS. The relative abundance *of Bacteroides*, which typically increases in patients with IBD^60^ and in healthy or CD smokers^66^, decreased following CS exposure in DSS-treated mice. In contrast, but in line with previous observations in humans^60,67,68^, mice not treated with DSS but exposed to CS for four weeks had increased *Bacteroides* relative abundance compared with Sham-exposed mice. Finally, CS exposure decreased the relative abundance of genera such as *Alistipes*, *Muribaculum*, and *Barnesiella*, all belonging to class Bacteroidia, in both DSS-treated and untreated mice.

Moreover, the abundance of *Lactobacillus* decreased in a CS-dependent manner at both post-DSS and recovery time points and for both DSS-treated and untreated mice. *Lactobacillus* has been suggested to exert anti-inflammatory effects by degrading selective cytokines^69^ or binding to a specific receptor, macrophage galactose-type lectin 1, in the intestine^70^. Clinical studies have also shown that *Lactobacillus* organisms decrease in the intestinal microbiota of IBD patients^71^ and may be effective in the management of UC^72^. These reports appear to conflict with our data. However, it is worth noting that the *Lactobacillus* species are phylogenetically diverse, with more than 100 documented to date^73^. Species-specific *Lactobacillus* effects have been described in a DSS-induced colitis mouse model^74^ and *ex vivo* cultured human tissues^75^. Thus, treatment with *L. paracasei* can delay the development of colitis and decrease its severity, but *L. plantarum* and *L. rhamnosus GG* can exacerbate disease development^74^. Finally, these three *Lactobacillus* species were shown to exert adverse effects on inflamed tissue derived from IBD patients and cultured *ex vivo*, while the supernatant from *L. paracasei* culture system downregulated the pro-inflammatory activities of leukocytes^75^. These observations underscore that a study of the role of *Lactobacillus* in IBD development must be species-specific, and detecting *Lactobacillus* species with high confidence is not fully supported by the shotgun sequencing method we used.

Finally, the most striking effect observed in our study was the CS-dependent increase in the abundance of *Akkermansia*, an anaerobic Gram-negative genus identified as a key mucin degrader^76^. Initially isolated from human fecal samples, *Akkermansia* species are commonly found in the human gut with variable abundance and are present both in feces and at the mucosal surface. *Akkermansia* is substantially reduced in the mucosal tissues of CD and UC patients^77^, and its abundance is inversely correlated with the onset of inflammation in metabolic diseases^29^. Further support for the beneficial effects of *Akkermansia* on colitis is derived from the observation that *Akkermansia* extracellular vesicles protect against DSS-induced colitis^78^ and from *Akkermansia’s* ability to produce propionate^79^,a possible anti-inflammatory agent in the gut^80^. Nonetheless, several publications have reported increased *Akkermansia* abundance in mice following DSS treatment^27,61,79^. The varying effects of inflammation on mucin expression and mucus production may explain these contradictory findings. Regardless of the causative agent, lower levels of inflammation stimulate mucin production, while high levels inhibit it^79^. In our model, the expression of several mucin genes was consistently upregulated by DSS treatment, explaining the increase in *Akkermansia* in the DSS-treated group that was not exposed to CS. CS alone appeared to upregulate *Muc5b*, as observed previously in other tissues^81^, while reducing the expression of *Muc4*. However, when DSS-treated mice were exposed to CS, the effect on mucin gene expression was relatively low and consisted mainly of downregulating *Muc13*, *Muc16*, *Muc4*, and *Muc1* during the recovery time point. This renders the strong, additive, CS-mediated *Akkermansia* increase observed at the post-DSS time point not linkable and difficult to explain.

The association between CS, gut microbiota, and IBD is receiving increased attention^34,58,67,68^. We found that CS exposure affects both the immune system and gut microbiota, contributing to the creation of a protective environment that may explain the observed CS-associated reduction in colitis severity. While the interplay between these parameters is unclear, we can speculate regarding the interaction between CS and colon physiology. First, CS or one of its constituents may directly affect the intestinal immune system, reducing inflammation and thus protecting from DSS-induced colitis. The observed changes in the gut microbiota would therefore only be a consequence of the CS-dependent reduction of inflammation or gut damage. This hypothesis would be supported by several publications showing a CS-dependent shift in the intestinal immune cell composition^16,19,49^, nicotine activation of the cholinergic anti-inflammatory pathway^48^, or CS-dependent decreases of NF-κB-p65 phosphorylation^63^ and cytokine level alteration^34^. Second, the observed CS-mediated changes in the gut microbiota may be the leading cause of the reduction in inflammation and colitis severity. The effect of smoking on gut microbial composition and diversity has been demonstrated in a number of pre-clinical and clinical studies^58^, linking those changes to both positive and negative effects of CS in UC and CD, respectively. Here we observed a striking CS-dependent modulation of genera such as *Akkermansia*, *Intestinimonas*, and *Lactobacillus*, whose metabolic products (e.g., short-chain fatty acids, propionate) may create a favorable intestinal milieu, protecting from DSS-induced inflammation or boosting recovery.

An important limitation of the study is that we focused on the whole affected organ, the colon, without focusing on the different cell types that populate this region during inflammation. Hence, more work is needed to identify the detailed mechanisms by which CS affects UC severity. In fact, it is likely that neither immune modulation nor microbial changes are the sole drivers, but rather players in a complex chain of interactions leading to the attenuation of the UC severity by CS. While the insights from the current study cannot be directly translated into the human condition, we tried to reflect a human real-life condition as much as possible. Thus, we used a well-established UC mouse model such as the DSS model, we exposed the mice to translatable human equivalent doses of CS, and finally, using omics and computational approaches, we placed the observed molecular changes into biological signaling pathways.

The risks of smoking far outweigh any possible benefit, thus smoking cessation or any other complementary approaches, as suggested by the tobacco harm reduction initiative^8,9^, to accelerate the decline in smoking prevalence and hence the smoking-related population, must be the first step toward well-being and a healthy life. However, the inverse correlation between CS and UC cannot be ignored and the role of certain compounds of tobacco when decoupled from combustion needs to be further addressed. Thus, in line with tobacco harm reduction principles^8,9^, further investigation is needed to identify the molecular mechanisms that may represent new targets in customized therapeutics or probiotics.

## Materials and Methods

### Animals

All procedures involving animals were performed in an Association for Assessment and Accreditation of Laboratory Animal Care International-accredited, Agri-Food & Veterinary Authority of Singapore-licensed facility at Philip Morris International Research Laboratories (PMIRL; Singapore). Care and use of animals were in accordance with guidelines set by the National Advisory Committee for Laboratory Animal Research in 2004^82^. All protocols were approved by the Animal Care and Use Committee of PMIRL (IACUC protocol #15049) in compliance with the National Advisory Committee for Laboratory Animal Research Guidelines on the Care and Use of Animals for Scientific Purposes^82^. Male C57Bl/6 mice (8–10 weeks of age) were obtained from In Vivos. Pte. Ltd. (Singapore), housed under specific hygiene conditions in filtered, conditioned fresh air at 22 ± 2 °C and 55% ± 15% humidity, and kept on a 12-hour/12-hour light/dark cycle.

Cage enrichment (Igloo™, Biosys Corp. PTE Ltd., Singapore, and Nylabone™, Neptune City, NJ, USA) was provided in each cage during the non-exposure period. The bedding material (Lignocel® BK 8–15, J. Rettenmaier & Soehne, GmbH & Co KG., Rosenberg, Germany) consisted of autoclaved softwood (fir and spruce) granulate. A gamma-irradiated pellet diet (T2914C rodent diet, Harlan Laboratories, Indianapolis, IN, USA) and filtered tap water were provided *ad libitum* (food was not available during exposure). Mice were allocated to their respective treatment groups four days prior to the experimental start date using a randomization sequence based on body weight (Provantis, Instem, Stone, UK).

### Study design and DSS-induced colitis

Animals were exposed to CS or fresh air (Sham) for a total of 27 days. Colitis was induced by adding DSS to the drinking water on Days 15–20, followed by a seven-day recovery period (Days 21–27; no DSS, but continued CS or fresh air exposure) (Fig. 1B). DSS (molecular weight 40,000, TdB Consultancy, Uppsala, Sweden) was prepared to a concentration of 2.5% in filtered drinking water and provided to the animals on a daily basis. Control groups (no DSS) received drinking water from the same source. The general condition and health of the mice was monitored by routine measurement of body weight and periodic observation. As a humane endpoint, any animal losing more than 20% of its body weight (relative to weight at the start of DSS treatment) was euthanized. Water consumption was recorded daily by weighing the water bottles.

### Assessment of colitis

Body weight was measured daily during the DSS treatment period and subsequent recovery period and examined as gross body weight as well as relative to baseline (pre-DSS treatment) body weight. On Day 4 post-DSS, stool samples were collected and evaluated for occult blood using hemoccult blood test strips (Hemoccult II Dispensepak Plus Test, Beckman-Coulter, Brea, CA, USA). Scoring was conducted according to the manufacturer’s procedures and used to calculate the disease activity index (DAI) (Table 2). Necropsy was performed after the seven-day recovery period. Animals were anesthetized with pentobarbital (100 mg/kg, i.p.) and exsanguinated via perfusion with 0.9% NaCl solution. The colon was isolated by trimming at the ileocecal junction and the distal end of the rectum and measured for total length. Colon contents were removed, and the colon was weighed before partitioning for molecular analysis. The DAI was a composite score derived from a compilation of relative body weight loss, change to the colon length/weight ratio, and occult blood in the stool measurements^16^. These parameters were tabulated and consolidated as described in Table 2.

**Table 2:** Scoring system for determining the DAI.

Group	Weight loss	Weight length	Haemoccult (blue coloration)
0	0–5%	<40	normal
1	5–10%	40–45	Very faint
2	10–15%	45–50	Faint
3	15–20%	>50	moderate
4	>20%		excessive

### Transcriptomic data analysis

#### Statistical models

Statistical models for analyzing the transcriptome data followed the study design, which consisted of one time point (end of the recovery period) and a combination of two factors (DSS and three-tier CS treatments). In the first stage, we restricted ourselves to seven interrelated pairwise comparisons to a common reference (control for DSS, and Sham for CS). The aim of these seven comparisons was to capture the effects of DSS treatment, the effects of the exposure to the three CS concentrations, and the combined effects of DSS and of the three CS concentrations. In the second stage, we specifically focused on how the three-tier CS exposure modulated the effects of DSS treatment, as drug treatments modify the effects of a disease. To capture this specific contribution, we needed to compare two effects obtained from pairwise comparisons (i.e., (DSS vs. control)_CS_ vs. (DSS vs. control)_sham_). This led to the “difference of differences” contrasts (i.e., ([DSS + CS] − [control+CS]) − ([DSS + sham] − [control + sham])), or the two-factor interaction effect in the language of experimental design (Supplementary Fig. S5). Adequate additive statistical models capturing these effects into suitable contrasts were fitted to the normalized transcriptomics data using the *limma* package^83^. The *p*-values for each contrast were adjusted across genes using the Benjamini-Hochberg false discovery rate (FDR) method. Differentially expressed genes (DEG) were defined as genes with FDR < 0.05.

#### Ingenuity® pathway analysis (IPA)

The results of the *limma* fits were subjected to IPA and upstream regulator (UR) analysis to assess the linkage to DEGs through coordinated expression, identifying potential URs such as transcription factors and any gene or small molecule that has been observed experimentally to affect gene expression directly or indirectly^26^. The resulting URs were graphed using the association confidence score provided by StringDB (https://string-db.org/)^84^.

#### Network perturbation amplitude (NPA)

The NPA methodology has previously been applied successfully in the context of UC^20^. Here, the dedicated toll-like receptor (TLR)-interleukin (IL)-1 receptor (IL1R)-tumor necrosis factor (TNF) receptor (TNFR) network model and the quantitative NPA approach were used to evaluate the DSS effect captured by the pairwise comparisons. Instead of assuming that the gene expression changes result in changes in protein activity, the network approach converts the fold changes in gene expression into predictions of activation or inhibition of network model entities that are known to regulate the expression of these genes (Fig. 3E)^20^.

#### Gene-set analysis (GSA)

GSA does not depend on fixed gene-level statistical significance thresholds (e.g., *p*-value thresholds), supporting more robust quantitative comparisons across conditions^85^. We used GSA to characterize how the three-tier CS exposure modulated the effects of DSS treatment. The effect of DSS treatment in the colon was greater than that of CS (Fig. 3A), so investigating their mutual influence was best captured using a highly sensitive two-factor interaction model. The three “difference of difference” contrasts were used in a gene set Q1 enrichment analysis based on the Reactome gene set collection^86^. Reactome was selected as an appropriate open-access gene set collection because of its comprehensive coverage of immune-related pathways (more than 500 gene sets, more than 120 of which are immune system-related). To preserve the high sensitivity of the results, we omitted the multiple-testing correction. This was justified by the significant overlaps among Reactome gene sets, violating the independence assumption used in multiple-testing corrections. To control the specificity of the results, we performed an over-representation analysis of the gene sets in terms of the top pathway categories provided by Reactome. The fact that the gene sets were distributed into top pathway categories in a non-random manner confirmed the validity of the results. We used the Reactome hierarchy to assign every gene set a top category and used the over-representation statistics implemented in the *piano* package to calculate the statistics^87^. We also used the concept of “leading-edge genes” to extract the subset of pathway genes that most contributed to an enrichment score^85^.

### Microbiome bioinformatics

Sequencing reads obtained from the previous step were cleaned of adapters using bbduk^88^ (adapter stripping, options k = 17, mink=8, ktrim=r, qin=33, minlength=100) and trimmed to a maximum length of 150 bases. After adapter cleaning, the reads were mapped using the Minimap2 software^89^, in sequence, to the mouse genome (GRCm38), the human genome (GRCh38), a viral sequences collection, and a collection of plasmid sequences. At each step, only sequences for which neither read could be mapped were retained. After cleaning, the reads were mapped to a reference database of microbial genomic sequences. This microbial database was formatted using minimap2, and the reads were mapped against this database. Reads flagged as “properly paired” that had a mapping quality ≥ 5 were retained. Reads were counted per sequence, resulting in one vector of counts per sample, and counts were merged into a single matrix. Two samples with fewer than 100,000 raw reads were removed. Sequences were assigned to taxa based on the NCBI taxonomy; the read count for a given taxon at any taxonomic rank was the number of reads assigned to it or any of its child taxa. Raw read counts were normalized using Deseq. 2^90^. Initially, the species-level normalized read counts were sorted, and the most abundant species, which together accounted for up to 98% of the total, were selected for further analysis: all sequences belonging to these species were selected, and a new raw read count matrix was generated. This set of filtered read counts was used to generate normalized genus-level abundances; this set was used in all subsequent analyses. The Shannon index was calculated using the R package *vegan*^91^ by first rarifying the raw read counts to the lowest common read count with the “rrarefy” function and then calculating the respective index with the “diversity” function. Boxplots were generated using ggplot2 from the subset of samples at time points “Post-DSS” and “Recovery.” The multivariate structure of the microbial database was investigated, as it was composed of various bacterial species identified from their derived normalized read counts. In the presence of a known group structure (provided by the study design factors, such as DSS and smoke exposure), we searched for linear combinations of bacterial species that allow the best discrimination between the study groups. To that end, we transformed the original variables (bacterial species) to their canonical variates, resulting from the maximization of the between-groups to the within-groups variance ratio. Biplots of the canonical variate analysis are given in Fig. 5B. Differential abundance testing was performed using Deseq. 2: for each time point (“Post-DSS” and “Recovery”), the four DSS/exposure combinations were compared to the water/Sham exposure group, and for each contrast, the differential abundance values for the 20 most abundant taxa were graphed.

### Statistical evaluation

The statistical evaluation consisted of performing targeted group comparisons across the study outcomes based on common hypotheses of interest. The statistical comparisons were performed for a given treatment period and, consequently, for a given animal cohort; this is true for all endpoints other than body weight and water consumption, where repeated measurements were registered for each animal. Comparisons were made using either a two-sample *t*-test for mean differences between two treatment groups of interest or by testing the significance of the appropriate coefficient within a statistical linear model. The latter enabled testing of the main effects of CS and DSS as well as their two-way interaction. Depending upon the endpoint under consideration, *p*-values were adjusted to address the high-dimensional aspect present in many analyses, notably with the transcriptome data. More information on statistical treatment for each analysis category is detailed in the preceding paragraphs. Datasets, further detail on the protocols, and additional data visualizations are available on the INTERVALS platform at 10.26126/intervals.q58qcf.1.

## Supplementary information

Supplementary Information.
