# Supplementary material for: The reduction of DSS-induced colitis severity in mice exposed to cigarette smoke is linked to immune modulation and microbial shifts
Source: Sci Rep. 2020 Mar 2;10:3829. doi: 10.1038/s41598-020-60175-3 (PMC7052152; doi:10.1038/s41598-020-60175-3)
Supplement: Supplementary file 1 — Supplementary Information. [file 41598_2020_60175_MOESM1_ESM.docx]

The reduction of DSS-induced colitis severity in mice exposed to cigarette smoke is linked to immune modulation and microbial shifts

Giuseppe Lo Sasso^1,3^, Blaine W. Phillips^2,3^, Alain Sewer^1^, James N.D. Battey^1^, Athanasios Kondylis^1^, Marja Talikka^1^, Bjoern Titz^1^, Emmanuel Guedj^1^, Dariusz Peric^1^, David Bornand^1^, Remi Dulize^1^, Celine Merg^1^, Maica Corciulo^1^, Sonia Ouadi^1^, Rendy Yanuar^2^, Ching Keong Tung^2^, Nikolai V. Ivanov^1^, Manuel C. Peitsch^1^, Julia Hoeng^1,*^

^1^ PMI R&D, Philip Morris Products S.A., Quai Jeanrenaud 5, CH-2000 Neuchâtel, Switzerland

^2^ PMI R&D, Philip Morris International Research Laboratories Pte. Ltd., Science Park II, Singapore

^3^Equally contributed

**Short title: Cigarette smoke reduces colitis severity in mice**

**^*^Corresponding author:**

Julia Hoeng

Global Head of System Toxicology

PMI R&D, Philip Morris Products S.A.

Quai Jeanrenaud 5

2000 Neuchatel, Switzerland

Phone: +41 (58) 242 2892

Email: [Julia.Hoeng@pmi.com](mailto:Julia.Hoeng@pmi.com)

# Supplementary material

# Supplementary Material and Methods

## Aerosol generation and exposure

Mice were whole-body exposed to diluted mainstream CS from research-grade 3R4F reference cigarettes for a total of four hours per day (Figure 1A). Exposures were performed to three target concentrations of total particulate matter (TPM): CS Low (450 µg/L), CS Medium (600 µg/L), and CS High (750 µg/L) ^1^. An intermittent exposure regime was used in which one-hour exposure blocks were interrupted with fresh air periods for 30 minutes after the first hour of smoke exposure and for 60 minutes after the second and third hours of exposure to avoid a build-up of excessive carboxyhemoglobin (COHb) concentrations in the 3R4F groups. The 3R4F cigarettes were purchased from the University of Kentucky (Kentucky, USA)^2^. Cigarettes were conditioned at 22 ± 1˚C and 60 ± 3% relative humidity for two to seven days prior to use.

Exposure was conducted in whole-body inhalation exposure chambers five days per week (seven days per week during the DSS treatment and recovery period) to the target mainstream CS concentration at each exposure chamber’s breathing zone. These CS exposure concentrations and durations have been shown to be well tolerated by C57Bl/6 mice in previous studies ^1^.

The test atmosphere was generated in basic conformity with the Health Canada Intense Smoking Protocol ^3^ (i.e., two-second puff duration, 55 mL puff volume, and puff frequency of two puffs per 60 seconds), with some minor deviations necessary for technical reasons (e.g., smoking whole puffs instead of rounding to the nearest tenth of a puff). 3R4F CS was generated on 30-port rotary smoking machines with active sidestream exhaust (type PMRL-G, SM2000) and equipped with a programmable dual-port syringe pump, as previously described ^4^. The mainstream CS was conveyed via glass tubing from the smoking machine to the exposure chamber. The average flow rates in the exposure chambers ranged from 132.0 L/minute to 137.6 L/minute. The inlet temperature of the test atmosphere in the exposure chamber was consistently maintained within the temperature and relative humidity ranges of 19.0–25.0°C and 30–70%, respectively. Air flow through the exposure chamber was monitored continuously during the exposure period. 3R4F cigarettes were smoked to a butt length of 35 mm. Static burning rate ranged from 428 seconds to 471 seconds, with a 10–11 puff count per stick. Human equivalent dose calculation: the above TPM concentrations corresponded to nicotine concentrations in the test atmosphere of 40 µg/L, 32 µg/L, and 25 µg/L (Supplementary Table 1). Using a volume estimate of 27.3 mL/min and a 240-minute exposure time, this translates to a deposited dose of approximately 11.3 mg/kg, 9.0 mg/kg, and 7.1 mg/kg, calculated using the formula described by Alexander et al. ^5^, where the delivered dose is a function of the concentration multiplied by the respiratory minute volume and the exposure duration, divided by body weight. The human equivalent dose can be calculated from the body surface area using a correction factor of 12.3 (mouse-to-human conversion for human equivalent dose using mg/kg). This translates to 64.4 mg, 51.1 mg, and 40.6 mg of delivered nicotine, corresponding to 46, 37, or 29 cigarette sticks (assuming 1.39 mg nicotine per stick).

## Analytical characterization of the test atmosphere

The stability and reproducibility of the test atmospheres were determined through systematic evaluation of the TPM concentration at the breathing zone (four times per day), nicotine (four times per day), carbon monoxide and aldehydes (weekly), and particle size distribution (once per chamber) (Supplementary Table 1). Relative humidity was measured only in the Sham exposure chamber, owing to interference with the particulates in the CS which damages the humidity sensor. All evaluations were performed according to previously described analytical methods ^6^.

## Analysis of CS and nicotine biomarkers

Biomarkers of exposure were determined to validate uptake of specific components of the test atmosphere by analyzing blood COHb and nicotine and cotinine concentrations in plasma. Blood for all bioanalytical procedures was collected during exposure week 2 (before DSS administration). The blood was collected from the facial vein of isoflurane-anesthetized animals within 20 minutes of removal from the exposure. Blood COHb levels were measured using the Cobas blood gas system (Roche, Basel, Switzerland). Nicotine and cotinine were measured from plasma samples using liquid chromatography–tandem mass spectrometry-based methods (Analytisch-biologisches forschungslabor GmbH, ABF, Munich, Germany).

## Histopathology

At necropsy, tissue samples (approximately 1.0 cm) were trimmed from the proximal and distal colon for molecular analysis (RNA and protein). The remaining portion of the colon was prepared for histological evaluation using Swiss roll embedding starting from the distal end (in the center of the roll). The tissue was fixed for approximately 24 hours in 4% formaldehyde and stored in ethanol (50%). The samples were processed using a standard ascending ethanol series, followed by xylene substitute and molten paraffin embedding. Sections were cut at 5 µm thickness, then adjacent sections were stained for hematoxylin and eosin and periodic acid Schiff stains respectively.

Slides were sent to a board certified Pathologist for histopathological evaluation (Advanced Molecular Pathology Lab (AMPL), Singapore). The samples were evaluated in a fully blinded manner (animal numbers provided using coded numbering). Evaluation was conducted for the following criterion: Severity of inflammation (0, normal mucosa; 1, mild inflammation; 2, moderate inflammation; 3 severe inflammation), Ulceration (0, absent; 1, present), Area of inflammation (0, 0%; 1, 1-25%; 2, 26-50%; 3, 51-75%; 4, 76-100%), and Hyperplasia and dysplasia (0, normal mucosa; 1, mild dysplasia; 2, low-grade dysplasia; 3, high-grade dysplasia).

## RNA isolation and transcriptomic data generation

Total RNA was isolated from colon samples. The RNA integrity number values of the samples (three experimental repetitions) was evaluated, and only samples with RNA integrity numbers ≥ 6 were processed further. One hundred nanograms of total RNA were reverse-transcribed to cDNA using an Affymetrix® HT 3′-IVT PLUS kit (Affymetrix, Santa Clara, CA, USA). The cDNA was labelled and amplified to complementary RNA (cRNA). The fragmented and labelled cRNA was hybridized to a GeneChip® Mouse Genome 430 2.0 Array (Affymetrix) in a GeneChip® Hybridization Oven 645 (Affymetrix) according to the manufacturer’s instructions. Arrays were rinsed and stained on a GeneChip® FS450 DX Fluidics Station (Affymetrix) using the Affymetrix® GeneChip® Command Console® software (AGCC software version 3.2, protocol FS450_0001). The arrays were scanned using a 3000 7G GeneChip® Scanner (Affymetrix). Raw images from the scanner were saved as DAT files. The AGCC software automatically gridded the DAT file images and extracted probe cell intensities into CEL files.

## Transcriptomic data processing

The raw CEL files were processed using software packages from the Bioconductor suite of microarray analysis tools for the R statistical software environment ^7^. Background correction and frozen robust multiarray quantile normalization were applied to generate microarray expression values from the arrays that passed the quality controls (QC). The QC metrics examined the distributions of the log intensities, the normalized unscaled standard error, the relative log expression, and the median absolute value relative log expression, as well as the general aspect of the array pseudo- and raw images, and were obtained using the affyPLM package. Additionally, the Mouse4302_Mm_ENTREZG v16.0 Brainarray Custom CDF environment was used for probe set-level summary to obtain the normalized data matrix. The complete array data have been deposited in the ArrayExpress public repository (E-MATB-7988).

## Gene expression quantification by reverse transcription qPCR (RT-qPCR)

To confirm the results obtained by microarray transcriptomics, a panel of relevant RNA samples and genes were selected for quantification by the “gold standard” RT-qPCR technique. The selection contained 35 RNA samples and 19 genes, including three housekeeping genes, which are provided in the Supplementary table S3.

The Qiagen reverse transcription kit (RT2 First Strand Kit Cat. Number 330404) and the quantitative real-time PCR (SYBR Green ROX qPCR Mastermix Cat. Number 330521) were carried out according to the manufacturer’s instructions in a Viia7 instrument (ThermoFisher, MA,USA). Genomic DNA contamination was evaluated with Qiagen cat. 330011 RT² qPCR Primer gDNA Control kit. Ct values generated with with the QuantStudio software Version 1.2 (with automatic baseline and threshold set to 0,2) were reported as arithmetic means of three technical replications of the amplification process and imported into the R statistical software environment for final processing. Using the three housekeeping genes (Actb, B2m, and GusB), the expression levels (δCt) were calculated by the mean-based normalization implemented in the Bioconductor NormqPCR package ^8^. The differential expressions were obtained using the same statistical models as in transcriptomics (pairwise comparisons and “difference of differences” contrasts), with the difference that t-statistics was used for calculating the p-values (instead of moderated t-statistics).

## Fecal sample processing and sequencing

To evaluate changes in the microbiota, stool samples were collected from individual animals in a longitudinal manner post-DSS treatment and on the day of necropsy (recovery). Samples were then stored at −80°C until further processing. DNA was extracted from fecal samples using a ZymoBIOMICS DNA Miniprep kit (cat. no. D4300; Zymo Research, CA, USA) and protocol ver. 1.2.2. The samples were ground using a MagNA Tissue Lyser (Roche), and the extracted DNA was quantified on a Qubit 2.0 fluorometer (Thermo Fisher Scientific, MA, USA). The DNA was sheared using a Covaris E220 focused-ultrasonicator (Matthews, NC, USA), and DNA sequencing libraries were prepared using the NuGEN Ovation Ultralow system V2 kit (San Carlos, CA, USA). After pooling, the libraries were sequenced on an Illumina HiSeq 4000 (San Diego, CA, USA). The sequencing read data have been deposited in the European Nucleotide Archive public repository with the following accession number: PRJEB32790.

## qPCR analysis for key taxa

The relative abundances and fold changes determined by the shotgun DNA sequencing analysis were confirmed by qPCR for key taxa (see Supplementary Table S4 for taxa and the corresponding primers). From the post-treatment group, 4 sub-groups representing 36 DNA samples.

Briefly, 20 ng of DNA was used per well, using Powerup SybrGreen Cat: A25741 (ThermoFisher, MA, USA). The qPCR experiment was performed in the ThermoFisher Viia7 instrument according to the manufacturer's instructions. Negative (RNase free water) and positive control (Community DNA Standard Log distribution Zymo D6311 - Zymo, CA, USA) were used combined with Uni331-F and Uni797-R primer pairs. For each sample, the qPCR experiments were performed in triplicate and analyzed with the QuantStudio software (Version 1.2) with automatic baseline and threshold set to 0,2. Fla and Ali primers were excluded from the analysis as they were not expressed after 40 cycles. The Ct values were reported in a table for the downstream analysis.

From the remainder, the mean value was determined from the replicates; in the universal 16S primer group, outlier replicates were eliminated prior to averaging (3 samples has a single replicate with a Ct value > 30, whereas for the remainder, the Ct values were below 12). For each sample the taxon-specific relative abundances where calculated by taking the difference in Ct between the taxon-specific Ct and the Universal Ct, inverting the sign to negative, and by then taking the exponential using base 2.

## Multi-analyte profiling of colon cytokines

Production of granulocyte colony-stimulating factor (G-CSF), interferon (IFN) γ, IL-1α, IL-1β, IL-5, IL-6, IL-10, IL-12 (p40), IL-13, IL-17, keratinocyte chemoattractant, TNFα, IL-21, and IL-22 was analyzed from colonic tissue homogenates. Dissected tissues were homogenized in 450 μL of homogenization buffer containing phosphate-buffered saline (Thermo Fisher Scientific) supplemented with complete miniprotease inhibitor cocktail (Roche) and 10% fetal calf serum (Thermo Fisher Scientific). Samples were sonicated at 20% for 20 seconds using a sonifier (Branson, Danbury, CT, USA) and then centrifuged at 16,000 × *g* for 20 minutes at 4°C. The supernatant was transferred into new tubes and stored at −80°C. Cytokine levels were analyzed using xMAP technology (Luminex, Austin, TX, USA). Concentrations were determined using the MCYTOMAG-70K and MTH17MAG-47K commercial kits (MilliporeSigma, Burlington, MA, USA) according to the manufacturer’s instructions and subsequently analyzed with five-parameter curve fitting using FM3D (Luminex). The final concentrations were determined by inversely predicting (back-fitting) the median fluorescence intensities of the study test samples as provided by the instrument, using the five-parameter logistic curve:

$$f(x,A,B,C,D,E)=D+\frac{A-D}{{(1+{(x/C)}^{B})}^{E}}$$

with the five parameters $A, B, C, D, E$ being estimated by standard calibration curves repeated twice per instrumental run and including seven levels. The test samples were injected into the instrument at two dilutions, including two injections (technical replication) per sample and per dilution. After the appropriate dilution was selected, the two technical replicates were averaged, and one single quantification per study item (expressed in pg/μL) was subjected to statistical analysis.

## Supplementary figure S1


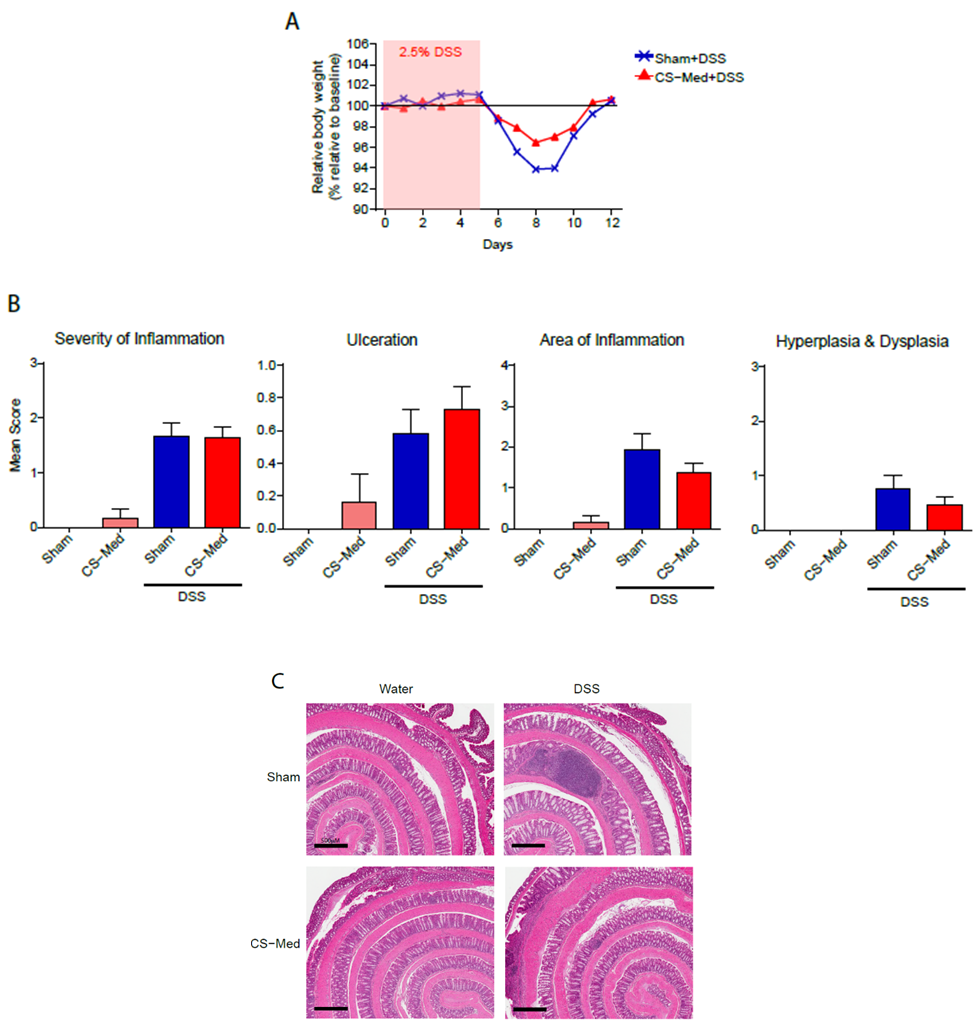


Histopathological assessment of Sham- and CS- (600 µg/L) exposed animals in the DSS-induced colitis model. **A**, Body weight progression was determined daily starting from the first day of DSS treatment (day 0) until the end of the recovery period (day 12 post-DSS). **B**, Pathological assessment scores were assessed for the depicted parameters following control, or DSS-treatment. **C**, Representative images of the H&E-stained colon slides are depicted (selected images were from samples near the median score for the parameter ‘area of inflammation’). Group sizes were n=6 (no DSS controls) and n=12 (DSS-treated groups). Results are presented as group mean (body weight, A) or mean ± SEM (B). Statistics were conducted, but no parameters reached p<0.05 compared to the respective Sham-treated group.

## Supplementary figure S2


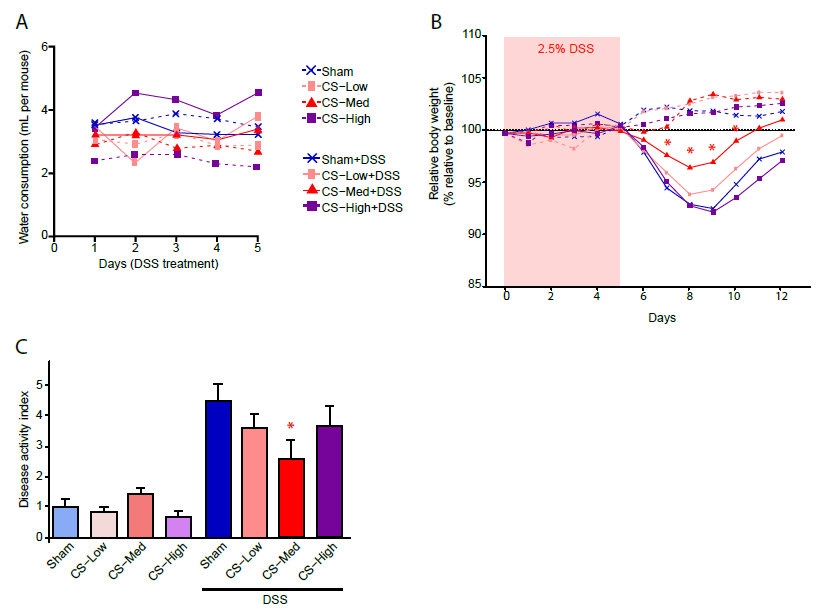


Effect of CS exposure (three concentrations) on clinical parameters of colitis induced by DSS. **A**, water consumption did not differ among the treatment groups. **B**, relative weight loss in treatment groups after removal of a single outlier in the medium (600 μg/L) CS group. **C**, DAI after removal of a single outlier in the medium (600 μg/L) CS group. DAI data are shown as means ±  SEM; **p* < 0.05. Only the mean is shown for A and B.

## Supplementary figure S3


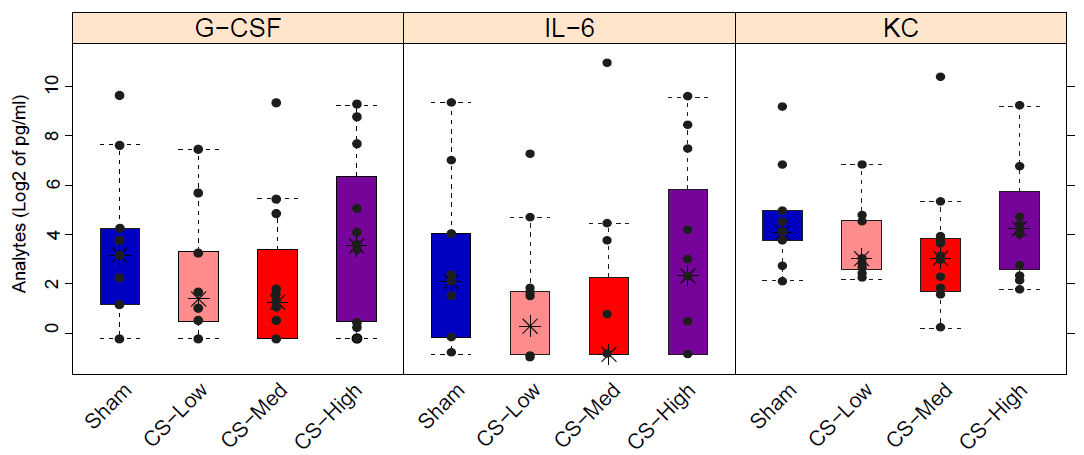


The abundance of selected cytokines in the distal colon of DSS-treated mice exposed to three concentrations of CS. Data are plotted on the log_2_ scale for three cytokines. The asterisks (*) represent the median.

## Supplementary figure S4


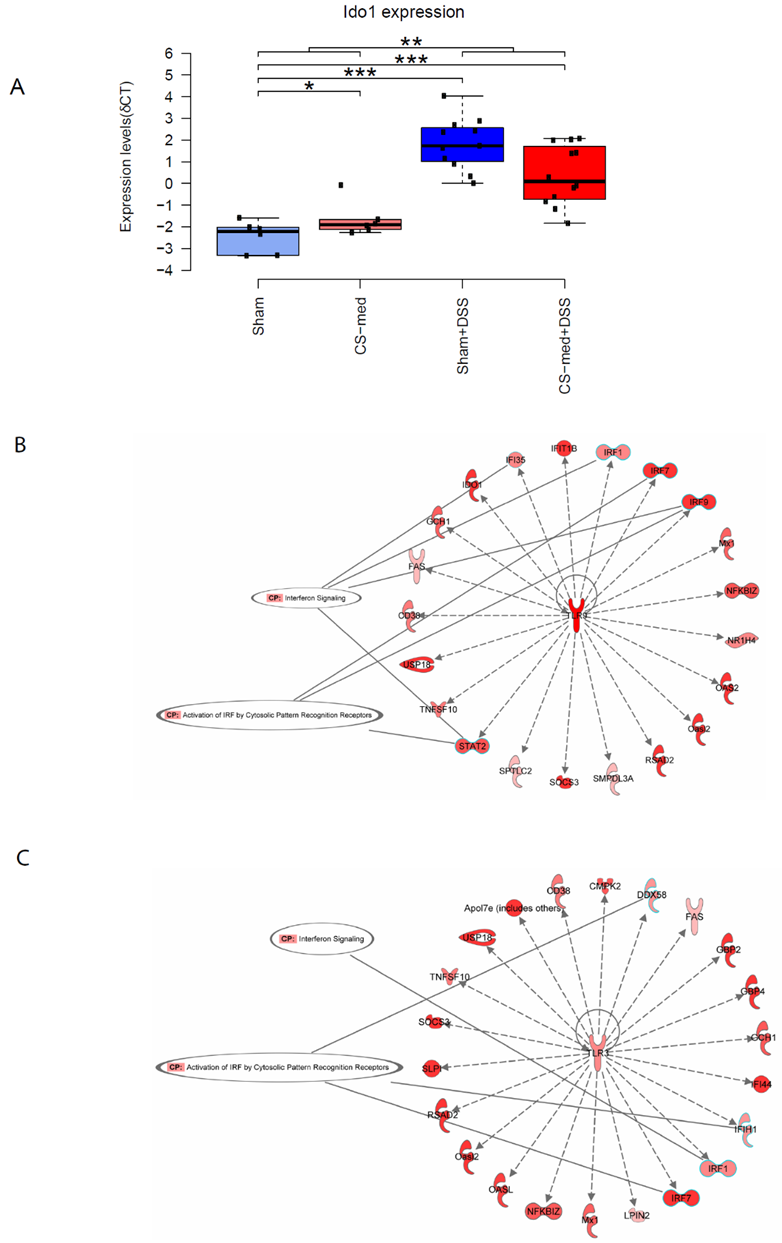


**A**. Box plot of the experimental group-based distributions of Ido1 expression levels in the mouse colon, as quantified by RT-qPCR. The horizontal brackets indicate the statistical significance of the corresponding comparisons or contrasts (*,**,*** mean p-value smaller than 0.05, 0.01, 0.001, respectively). The lower single brackets match the pairwise comparisons from Figure 3D, whereas the upper triple bracket matches the “difference of differences” contrast from Figure 4D.**B-C**. Ingenuity Pathway Analysis top two Canonical Pathways interaction with TLR9 (**A**) and TLR3 (**B**). The two canonical pathways (CP) “Activation of IRF by Cytosolic PRRs” and “Interferon Signaling” were identified by IPA as the most significantly upregulated in Sham+DSS group. TLR9 and TLR3, are linked to those two CP via specific genes, which are in turn upregulated in Sham+DSS group as well. None of these pathways is activated when mice are exposed to CS.

## Supplementary figure S5


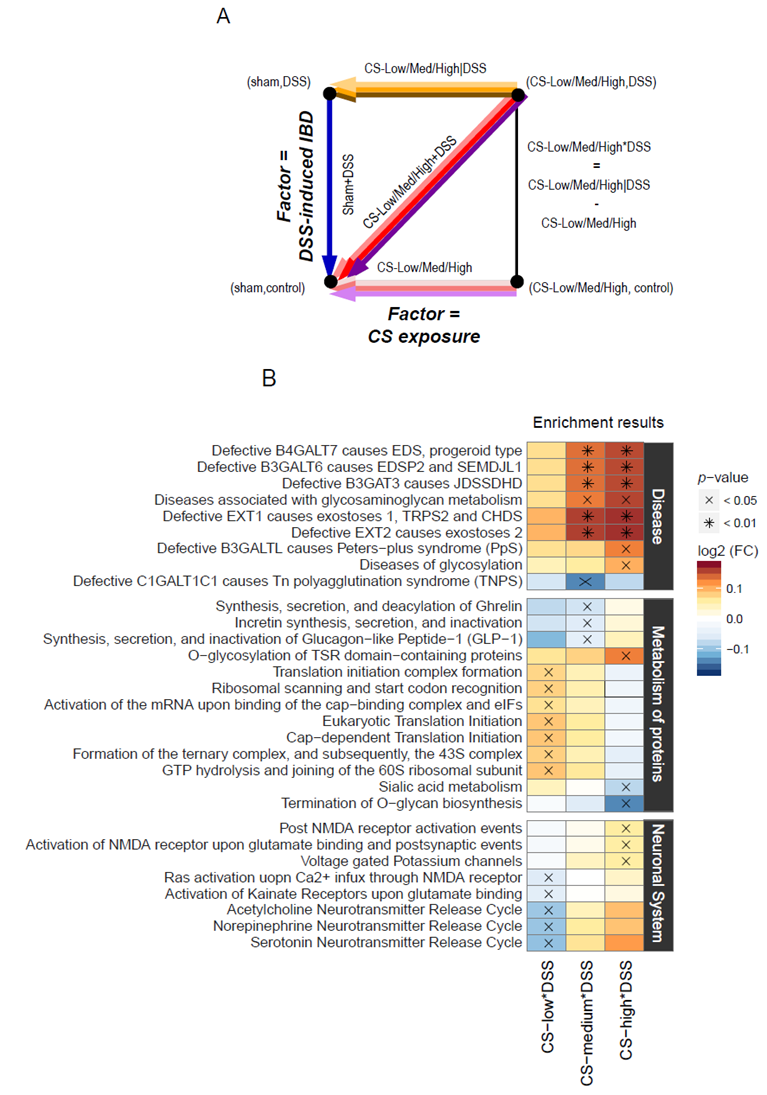


Overview of the statistical models used for transcriptomic and GSA for the modulating effects of CS exposure (three concentrations) on DSS-induced IBD. **A**, schematic representation of the two-dimensional experimental design space spanned by the two factors “DSS-induced IBD” and “CS exposure”. The points (●) represent the experimental groups characterized by the combinations of the two factors. The arrows describe the differential effects captured by the statistical models used in the analyses, e.g., the pairwise comparisons *sham+DSS*, *CS-Low/Med/High+DSS*, and the difference of differences/interaction *CS-Low/Med/High×DSS* = *(CS-Low/Med/High|DSS)−(CS-Low/Med/High)*. **B**, heatmap of gene set scores for the DSS treatment and CS exposure interaction, capturing the modulating effects of CS exposure on DSS-induced IBD. Only the pathways belonging to the over-represented Reactome categories “Disease, Metabolism of Protein”, and “Neuronal System” are shown. The annotations (X and *) indicate statistical significance by GSA Q1 raw *p*-values. NA, not applicable; FC, fold change.

## Supplementary figure S6


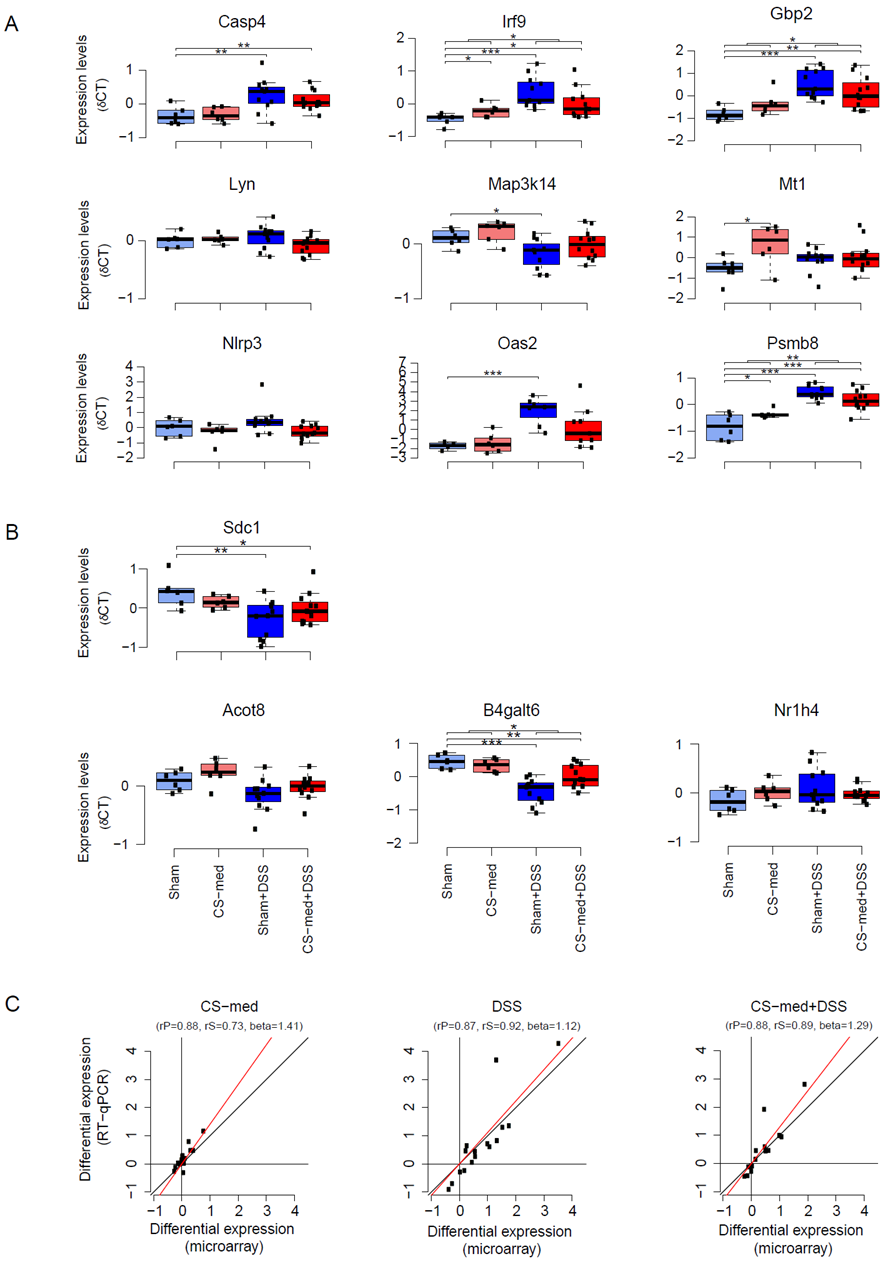


Confirmatory gene expression quantification by RT-qPCR. The same box plot conventions were used as in Supplementary figure S4A. **A**, box plots of the experimental group-based distributions of mouse colon expression levels for genes selected from the “Immune System” category (Figure 4D), as quantified by RT-qPCR. **B**, box plots of the experimental group-based distributions of mouse colon expression levels for genes selected from the “Metabolism” category (Figure 4D), as quantified by RT-qPCR. The horizontal brackets indicate the statistical significance of the corresponding comparisons or contrasts (*,**,*** mean p-value smaller than 0.05, 0.01, 0.001, respectively). The lower single brackets match the pairwise comparisons from Figure 3D, whereas the upper triple bracket matches the “difference of differences” contrast from Figure 4D. **C**, scatter plots comparing the mouse colon differential expression values obtained by microarray (horizontal axis) and RT-qPCR (vertical y-axis). Three following similarity metrics were used: “rP” is the Pearson correlation, “rS” is the Spearman rank-based correlation, and “beta” is the coefficient of a fitted intercept-free linear model. ).

## Supplementary figure S7


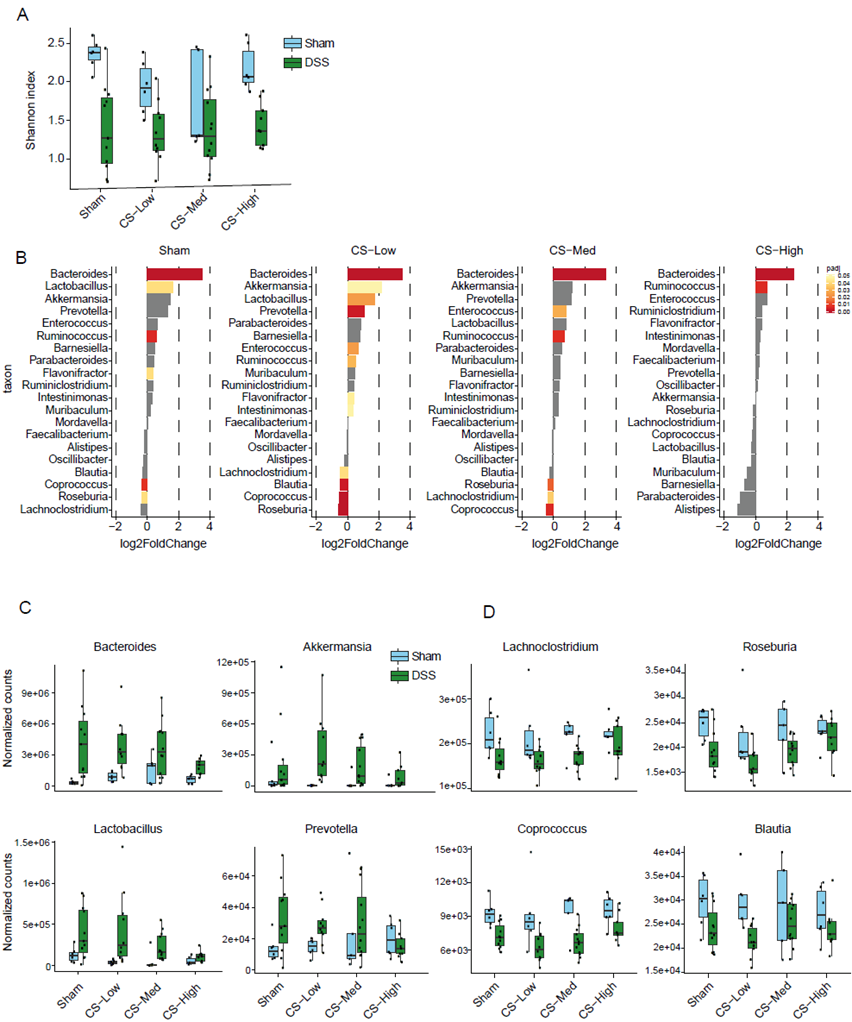


Results of metagenomics analyses following recovery from DSS treatment. **A**, alpha diversity (Shannon index) was calculated at the genus level and is displayed as a bar-and-whiskers plot for each individual combination of DSS treatment status and CS exposure after cessation of DSS treatment. The center line represents the median and the box encloses the 1st and 3rd quartiles (“hinges”). The upper and lower whiskers represent the furthermost points from the respective hinges, which are no more than 1.5 interquartile ranges from the hinge. The individual points are overlaid. **B**, differential abundance of bacterial genera for each combination of DSS treatment and CS exposure relative to the untreated and unexposed reference group (Sham). The top 20 most abundant genera determined by the Deseq2 BaseMean metric are reported for each comparison. Bars are colored by differential abundance (adjusted *p*-values [padj] < 0.05); grey bars: padj > 0.05. **C**, normalized read count for the four bacterial genera with greatest increases in abundance. **D**, normalized read count for the four bacterial genera with greatest decreases in abundance. The bar-and-whisker plots in D and E were constructed as described for A.

##
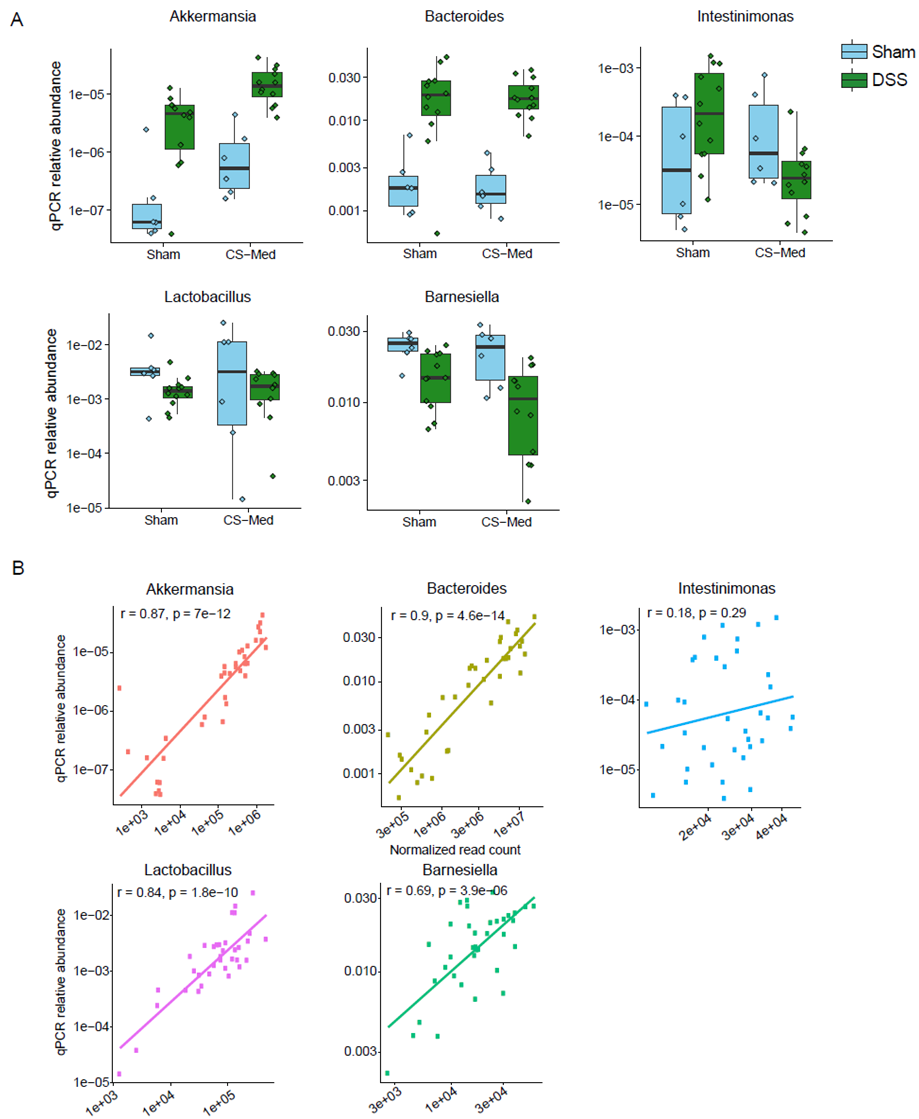
Supplementary figure S8

The abundance levels as determined by the Fold changes shotgun microbiome sequencing analysis were cross-checked using qPCR, based on 36 samples from the post-treatment time point. **A**, bars-and-whiskers plot (boxes, hinges and whiskers explained in the main part of the manuscript) of the scaled, relative abundance values for each taxon based on the qPCR data. **B**, correlation of the expression values from qPCR and from DNA-Seq for the samples at this time point. Please note that the display and analysis are performed in log-space.

## Supplementary Table S1

| **Test atmosphere** | **Exposure chamber** | **TPM (µg/l)** | **Carbon monoxide (ppm)** | **Nicotine (µg/L)** | **Formaldehyde (µg/L)** | **Acetaldehyde (µg/L)** | **Acrolein (µg/L)** | **MMAD**  **(µm)** | **GSD** |
| --- | --- | --- | --- | --- | --- | --- | --- | --- | --- |
| Fresh air | 1 | -2.6 ± 1.3 | 0.1 ± 0.0 | <LOD | 0.01 ± 0.01 | 0.02 ± 0.00 | <LOD | NA | NA |
| Cigarette Smoke  (low) | 2 | 454.4 ± 8.1 | 513.4 ± 11.8 | 24.7 ± 1.8 | 0.63 ± 0.07 | 28.73 ± 0.67 | 2.46 ± 0.05 | 0.82 | 2.51 |
| Cigarette Smoke (medium) | 3 | 591.8 ± 30.8 | 653.1 ± 30.7 | 32.2 ± 3.7 | 0.76 ± 0.07 | 39.02 ± 3.79 | 3.28 ± 0.31 | 0.85 | 1.62 |
| Cigarette Smoke  (high) | 4 | 744.6 ± 23.7 | 802.8 ± 24.8 | 40.0 ± 2.4 | 0.73 ± 0.10 | 43.24 ± 4.77 | 3.64 ± 0.36 | 0.83 | 1.35 |

Characterization of test atmosphere in the exposure chambers

Test atmospheres were generated in basic conformity with the Health Canada Intense Smoking Protocol. Data are shown as means  ±  SD. In the sham-exposure (fresh air) chamber, all measurements were marginal (below the LOD) or undetectable (NA). TPM, total particulate matter; MMAD, mass median aerodynamic diameter; GSD, geometric standard deviation.

## Supplementary Table S2

Cytokine values following exposure to three cigarette smoke (CS) concentrations and dextran sulfate sodium (DSS) treatment relative to levels in controls

|  | **No DSS treatment** | **DSS treatment** | | | |  |  |
| --- | --- | --- | --- | --- | --- | --- | --- |
| Cytokine | Sham | Sham | CS-Low | CS-Med | CS-High | LOD | LOQ |
| G-CSF | -0.23 (0) | 3.83 (1.07) | 2.31 (0.82) | 2.13 (0.87) | 3.5 (1.13) | 0.77 | 1.43 |
| *IFN* | *-0.86 (0)* | *1.36 (1.01)* | *-0.6 (0.26)* | *-0.36 (0.29)* | *0.52 (1.05)* | *0.14* | *0.85* |
| IL-10 | 0.37 (0.25) | 0.85 (0.25) | 0.86 (0.27) | 0.48 (0.25) | 0.83 (0.3) | 1 | 1.68 |
| *IL-12p40* | *1.06 (0.1)* | *0.96 (0)* | *0.96 (0)* | *1.08 (0.12)* | *1.08 (0.11)* | *1.96* | *2.66* |
| *IL-13* | *1.96 (0)* | *1.96 (0)* | *1.96 (0)* | *1.96 (0)* | *1.96 (0)* | *2.96* | *4.32* |
| *IL-17α* | *3.22 (0)* | *3.57 (0.24)* | *3.22 (0)* | *3.36 (0.15)* | *3.5 (0.28)* | *4.22* | *4.85* |
| IL-1α | 3.19 (0.35) | 4.12 (0.5) | 3.65 (0.33) | 3.55 (0.41) | 4.42 (0.72) | 3.36 | 4.23 |
| IL-1b | 2.37 (0.33) | 2.7 (0.36) | 2.8 (0.27) | 2.77 (0.35) | 2.96 (0.59) | 2.43 | 3.29 |
| IL-21 | 5.43 (0.21) | 5.37 (0.16) | 5.56 (0.15) | 5.11 (0.16) | 5.42 (0.16) | 3.45 | 3.97 |
| IL-22 | 3.19 (0.16) | 3.89 (0.4) | 3.5 (0.24) | 3.35 (0.29) | 4.01 (0.57) | -1 | -0.74 |
| *IL-5* | *-0.67 (0.33)* | *-0.3 (0.27)* | *-0.17 (0.35)* | *0.13 (0.28)* | *-0.47 (0.25)* | *0* | *0.68* |
| IL-6 | -0.86 (0) | 2.9 (1.21) | 1.22 (0.89) | 1.08 (1.05) | 2.71 (1.18) | 0.14 | 1 |
| KC | 2.31 (0.31) | 4.69 (0.8) | 3.54 (0.45) | 3.38 (0.75) | 4.7 (0.72) | 1.2 | 2.17 |
| *TNFα* | *0.2 (0)* | *0.8 (0.35)* | *0.39 (0.19)* | *0.48 (0.21)* | *1 (0.53)* | *1.2* | *1.77* |

Values are means ± SEM (in parentheses), reported on the log_2_ scale. Italics indicate cytokines for which the majority of the quantified values were below the quantification or detection limits. Geometric means can be computed directly by raising the tabulated mean values to the power of two. LOD, limit of detection; LOQ, limit of quantification.

## Supplementary Table S3

Qiagen primer list for RT-qPCR quantification

| Product name | Product number | Gene name | Molecule Id number | Category |
| --- | --- | --- | --- | --- |
| RT² qPCR Primer Assay (200) | 330001 | Acot8 | PPM37101A | Metabolism |
| RT² qPCR Primer Assay (200) | 330001 | Actb | PPM02945B | Housekeeping |
| RT² qPCR Primer Assay (200) | 330001 | B4galt6 | PPM26869A | Metabolism |
| RT² qPCR Primer Assay (200) | 330001 | B2m | PPM03562A | Housekeeping |
| RT² qPCR Primer Assay (200) | 330001 | Casp4 | PPM03075C | Immune System |
| RT² qPCR Primer Assay (200) | 330001 | Gbp2 | PPM05998A | Immune System |
| RT² qPCR Primer Assay (200) | 330001 | GusB | PPM05490C | Housekeeping |
| RT² qPCR Primer Assay (200) | 330001 | Ido1 | PPM05363A | Upstream regulator |
| RT² qPCR Primer Assay (200) | 330001 | Irf9 | PPM04697C | Immune System |
| RT² qPCR Primer Assay (200) | 330001 | Lyn | PPM04034C | Immune System |
| RT² qPCR Primer Assay (200) | 330001 | Map3k14 | PPM03084A | Immune System |
| RT² qPCR Primer Assay (200) | 330001 | Mt1 | PPM06181G | Immune System |
| RT² qPCR Primer Assay (200) | 330001 | Muc1 | PPM03608A | Mucin |
| RT² qPCR Primer Assay (200) | 330001 | Muc2 | PPM24739G | Mucin |
| RT² qPCR Primer Assay (200) | 330001 | Nlrp3 | PPM29506F | Immune System |
| RT² qPCR Primer Assay (200) | 330001 | Nr1h4 | PPM24915A | Metabolism |
| RT² qPCR Primer Assay (200) | 330001 | Oas2 | PPM35813A | Immune System |
| RT² qPCR Primer Assay (200) | 330001 | Psmb8 | PPM32464A | Immune System |
| RT² qPCR Primer Assay (200) | 330001 | Sdc1 | PPM03216A | Metabolism |

## Supplementary Table S4

| Taxon specific primer | qPCR names | Sequence Forward | Sequence Reverse | Reference |
| --- | --- | --- | --- | --- |
| Akkermansia | Akk | CAGCACGTGAAGGTGGGGGACC | CCTTGCGGTTGGCTTCAGAT | Hakanson et al 2015 ^9^ |
| Bacteroides spp. | Bac1 | GCTCAACCKTAAAATTGCAGTTG | GCAATCGGRGTTCTTCGTG | Staudacher 2016 ^10^ |
| Bacteroides | Bac2 | GAGAGGAAGGTCCCCCAC | CGCTACTTGGCTGGTTCAG | Layton et al 2006 ^11^ |
| Flavonifractor plautii | Fla | GGTCGCATGGCTCTGACT | TCATTTGTTTCGTCCCCGAC | Alauzet et al 2019 ^12^ |
| Intestinimonas AF211 | Int | AAAACTATGGGCTCAACCCA | GTCAGTTAATGTCCAGCAGG | Bui et al 2015 ^13^ |
| Alistipes group | Ali | TTAGAGATGGGCATGCGTTGT | TGAATCCTCCGTATT | Roager et al ‎2014 ^14^ |
| Lact-F - Lact-R | Lac | AGCAGTAGGGAATCTTCCA | CACCGCTACACATGGAG | Hakanson et al 2015 ^9^ |
| Barnesiella | Bar | CCAAGTCGCGTGAAGGAAGA | ACGGAGTTAGCCGATGCTTT | Anhe et al 2019 ^15^ |
| Total bact (Universal) | Uni | TCCTACGGGAGGCAGCAGT | GGACTACCAGGGTATCTAATCCTGTT | Hakanson et al 2015 ^9^ |

Taxa and the corresponding primers

# References

1 Phillips, B. *et al.* A 7-month cigarette smoke inhalation study in C57BL/6 mice demonstrates reduced lung inflammation and emphysema following smoking cessation or aerosol exposure from a prototypic modified risk tobacco product. *Food and Chemical Toxicology* **80**, 328-345 (2015).

2 Roemer, E. *et al.* Mainstream smoke chemistry and in vitro and in vivo toxicity of the reference cigarettes 3R4F and 2R4F. *Beiträge zur Tabakforschung International/Contributions to Tobacco Research* **25**, 316-335 (2012).

3 Health Canada. (1999).

4 Phillips, B. *et al.* A six-month systems toxicology inhalation/cessation study in ApoE−/− mice to investigate cardiovascular and respiratory exposure effects of modified risk tobacco products, CHTP 1.2 and THS 2.2, compared with conventional cigarettes. *Food and Chemical Toxicology* (2019).

5 Alexander, D. J. *et al.* Association of Inhalation Toxicologists (AIT) working party recommendation for standard delivered dose calculation and expression in non-clinical aerosol inhalation toxicology studies with pharmaceuticals. *Inhalation toxicology* **20**, 1179-1189 (2008).

6 Stabbert, R. *et al.* Toxicological evaluation of an electrically heated cigarette. Part 2: Chemical composition of mainstream smoke. *Journal of applied toxicology : JAT* **23**, 329-339, doi:10.1002/jat.924 (2003).

7 R Core Team. (2018).

8 Perkins, J. R. *et al.* ReadqPCR and NormqPCR: R packages for the reading, quality checking and normalisation of RT-qPCR quantification cycle (Cq) data. *BMC genomics* **13**, 296, doi:10.1186/1471-2164-13-296 (2012).

9 Håkansson, Å. *et al.* Immunological alteration and changes of gut microbiota after dextran sulfate sodium (DSS) administration in mice. *Clinical and experimental medicine* **15**, 107-120 (2015).

10 Staudacher, H. M. *The impact of dietary interventions for irritable bowel syndrome on the gastrointestinal microbiota, symptoms, nutrient intake and quality of life*, King's College London, (2016).

11 Layton, A. *et al.* Development of Bacteroides 16S rRNA gene TaqMan-based real-time PCR assays for estimation of total, human, and bovine fecal pollution in water. *Appl. Environ. Microbiol.* **72**, 4214-4224 (2006).

12 Alauzet, C. *et al.* Hypergravity disrupts murine intestinal microbiota. *Scientific reports* **9**, 1-11 (2019).

13 Bui, T. P. N. *et al.* Production of butyrate from lysine and the Amadori product fructoselysine by a human gut commensal. *Nature Communications* **6**, 10062 (2015).

14 Roager, H. M., Licht, T. R., Poulsen, S. K., Larsen, T. M. & Bahl, M. I. Microbial enterotypes, inferred by the prevotella-to-bacteroides ratio, remained stable during a 6-month randomized controlled diet intervention with the new nordic diet. *Appl. Environ. Microbiol.* **80**, 1142-1149 (2014).

15 Anhê, F. F. *et al.* Treatment with camu camu (Myrciaria dubia) prevents obesity by altering the gut microbiota and increasing energy expenditure in diet-induced obese mice. *Gut* **68**, 453-464 (2019).
